# The HRAS-binding C2 domain of PLCη2 suppresses tumor‐like synoviocytes and experimental arthritis in rheumatoid arthritis

**DOI:** 10.1038/s12276-025-01393-5

**Published:** 2025-02-03

**Authors:** Hyun Min Jeon, Hae Sook Noh, Min-Gyu Jeon, Jin-Ho Park, Young-Sun Lee, Gyunghwa Seo, Yun-Hong Cheon, Mingyo Kim, Myung-Kwan Han, Jae-Yong Park, Sang-Il Lee

**Affiliations:** 1 Division of Rheumatology, Department of Internal Medicine and Institute of Medical Science, Gyeongsang National University School of Medicine and Gyeongsang National University Hospital, Jinju, South Korea; 2 School of Biosystem and Biomedical Science, College of Health Science, Korea University, Seoul, South Korea; 3 Department of Microbiology, Jeonbuk National University Medical School, Jeonju, South Korea

**Keywords:** Signal transduction, Rheumatoid arthritis

## Abstract

Fibroblast-like synoviocytes (FLSs), which are stromal cells that play key roles in rheumatoid arthritis (RA) pathophysiology, are characterized by a tumor-like phenotype and immunostimulatory actions. C2 domains in various proteins play roles in intracellular signaling and altering cellular characteristics, and some C2 domain-containing proteins exacerbate or alleviate certain malignant or inflammatory diseases. However, the roles of C2 domains in regulating the functions of RA FLSs remain unclear. Here we performed functional C2 domainomics with 144 C2 domain-containing viral vectors and identified the C2 domain of PLCη2 as a key regulator of RA FLSs. In mice, overexpressing PLCη2 or only its C2 domain PLCη2 (PLCη2_C2) diminished the proliferation, migration, invasion and inflammatory responses of RA FLSs, mitigating RA pathology; the absence of PLCη2 amplified these proinflammatory and destructive processes in RA FLSs in vivo. Mechanistically, PLCη2 and PLCη2_C2 participate in the pathological signaling of RA FLSs in a calcium-independent manner through protein–protein interactions. Specifically, PLCη2_C2 disrupted HRAS–RAF1 interactions, suppressing downstream signaling pathways, including the NF-κB, JAK–STAT and MAPK pathways. Collectively, these findings establish PLCη2 and PLCη2_C2 as novel inhibitory regulators in RA, suggesting promising therapeutic avenues for addressing FLS-driven disease mechanisms.

## PLCη2 C2 domain regulates inflammation in RA cells

Rheumatoid arthritis is a disease in which the immune system attacks the joints, causing pain and damage. Researchers are looking for new ways to treat RA by targeting specific cells in the joints called fibroblast-like synoviocytes. These cells behave like cancer cells and contribute to joint damage. Researchers studied a protein called PLCη2, which is less active in patients with RA. They used a method called high-throughput screening to find proteins that could be targeted to treat RA. They discovered that increasing PLCη2 levels in FLSs reduced their aggressive behavior and inflammation. The study involved experiments with human cells and mice. The researchers used viral vectors to increase PLCη2 in FLSs and observed reduced cell growth and increased cell death. They also found that PLCη2 interacts with another protein, HRAS, to block harmful signaling pathways.

This summary was initially drafted using artificial intelligence, then revised and fact-checked by the author.

## Introductions

Rheumatoid arthritis (RA) is a prototypical autoimmune disease that progresses through complex interactions between immune cells and stromal cells, leading to joint destruction and severe disability^1,2^. Despite rapid advancements in RA treatments (primarily immune cell-targeted drugs), a substantial proportion of patients continue to have refractory disease. In addition, many patients discontinue treatment owing to heightened risks for infections and malignancies associated with systemic immunosuppression^3,4^. Fibroblast-like synoviocytes (FLSs), the predominant joint-resident stromal cells, are instrumental in sustaining inflammation and advancing joint destruction in RA because they exhibit immune-activating roles and aggressive cancer cell-like behavior^1,3–7^. Our group has consistently focused on targeting RA FLSs because refractory RA probably depends on their persistent immunopathological activity within joints^8–13^. Furthermore, novel drugs that target RA FLSs may complement existing therapies by mitigating serious side effects through systemic immune suppression^4,5^.

A diverse array of proteins consists of combinations of different modules that are characterized by unique binding domains, such as the C2, SH2, PTB, PH, SH3, WW and PDZ domains^14,15^. Among them, C2 domains display remarkable abilities to bind various ligands and substrates, including calcium ions, phospholipids, inositol polyphosphates and intracellular proteins^16,17^. Currently, 233 distinct C2 domains have been identified in various proteins that play key roles in cellular signal transduction, membrane trafficking and structural support^16,17^. Interestingly, several unique C2 domain-containing proteins, such as double C2 domain protein B^18^, myoferlin^19^ and tandem C2 domain nuclear protein^20^, have been found to be differentially expressed in several cancer tissues and associated with tumor-promoting or tumor-suppressing functions. Therefore, methodical exploration of C2 domains is essential for understanding their pathogenic mechanisms and identifying new therapeutic targets for RA, where dysregulated proliferation and aberrant survival of RA FLSs strongly coincide with apoptotic resistance, increased aggressiveness and altered metabolic profiles of cancer cells.

Omics-analytical methods, including genomics, epigenomics, transcriptomics, proteomics and metabolomics, are used primarily to screen for genes and proteins that may serve as therapeutic targets, marking an initial step in new drug development. Selecting candidate molecules on the basis of quantitative mRNA or protein expression differences necessitates further validation of their functionality and disease associations. This process often combines in silico prediction tools, web-based platforms and both in vitro and in vivo experiments. However, these approaches can be time-consuming and costly and carry risks of failure^21,22^. By contrast, high-throughput screening (HTS)-based multiparametric cellular dynamic assays are becoming increasingly recognized for their ability to integrate and analyze both functional (for example, cell morphology, apoptosis, proliferation and differentiation) and molecular (for example, changes in protein levels, localization and intracellular phosphorylation) aspects from the onset of screening. This comprehensive strategy reduces the time required for selecting target molecules and increases the likelihood of successful drug development^23–25^. Previously, our team developed a novel HTS method based on C2 domainomics, which utilizes a multiparametric, cellular dynamic assay^26^. This assay leverages a library of 144 C2 domain-containing viral vectors to screen for therapeutic targets within a vast array of C2 domain-containing proteins. Subsequently, we confirmed the utility of this innovative approach in studying the melanocyte differentiation and chondrogenic differentiation of human mesenchymal stem cells (MSCs)^27,28^. Uncontrolled differentiation resembles the proliferation and metastasis of melanoma^29^, a highly aggressive cancer whose incidence is exacerbated by immune dysregulation and the adverse effects of RA medications^30^. Furthermore, the prolonged inflammatory conditions of RA, associated with articular cartilage damage, suggest that MSCs (with potent anti-inflammatory and chondrogenic capacities) have high potential for successfully treating RA^31^. In addition, because RA FLSs share similarities with cancer cells and are derived from MSCs, they present overlapping molecular mechanisms and intracellular signaling pathways^7,31^. Therefore, this functional C2 domainomics-based HTS strategy, when applied to patient-derived RA FLS cell lines, holds substantial promise for identifying novel therapeutic targets for RA.

In this study, to identify a promising C2 domain-containing protein as a potential therapeutic target for RA, we screened the effects of our comprehensive C2 domain-containing viral vector library on cytokine-stimulated proliferation and NF-κB activation in RA FLSs. We checked the levels of the candidate protein in the synovial tissue and FLSs of patients with RA. We also explored the roles of the target C2 domain-containing protein using RA FLSs and several experimental mouse models. Finally, to clarify the mechanisms by which the candidate protein and its C2 domain act against RA, we evaluated its binding with calcium and protein‒protein interactions.

## Methods

### Preparing human synovial tissues and FLS lines and culturing cells

Each primary cell line was previously obtained from discarded synovial tissue from different patients with RA while undergoing synovectomy, as described previously^8–12^. All FLSs from passages 4–6 were used in the experiments. FLSs were maintained in Dulbecco’s modified Eagle medium (Gibco) supplemented with 10% fetal bovine serum (FBS) and 10% antibiotic–antimycotic solution (Thermo Fisher Scientific) at 37 °C in an atmosphere of 5% CO_2_, and they were used between passages 3 and 8. The MH7A synovial fibroblast cell line was obtained and cultured as described previously^8–12^. In brief, MH7A cells were cultured in RPMI 1640 (Gibco) supplemented with 10% heat-inactivated FBS, penicillin (final concentration, 100 U/ml) and streptomycin (final concentration, 0.1 mg/ml) at 37 °C in an atmosphere of 5% CO_2_ in air. All the cells were tested for mycoplasma contamination before their use in experiments and then synchronized in serum-starvation medium, such as IL-1β (10 ng/ml; R&D Systems, 201-LB), TNF (10 ng/ml; R&D Systems, 210-TA) or LPS (10 µg/ml; Sigma, L6529), for 12 h before the stimulus of interest was added. Because thapsigargin (TG) (2 µM and 5 µM; Sigma, T9033) induces apoptosis by inhibiting the endoplasmic reticulum (ER) Ca^2+^ ATPase pump, it was used for apoptosis induction in this study.

### Generating adenovirus constructs and plasmids and performing transfections

To construct recombinant adenoviral vectors encoding GFP-fused C2 domains, a C2 domain library was constructed using the sequences of 144 different C2 domains and the Gateway adenovirus system, as described previously^26–28^. In brief, 144 human C2 domain entry clones were obtained using Gateway cloning technology (Invitrogen). The resulting PCR products were inserted into the pENTR/D-TOPO vector (Invitrogen) and then subcloned into an adenovirus vector (pAdTrack-CMV) that expresses GFP under the control of the CMV promoter (that is, the pAd/CMV/V5-DEST vector; Invitrogen) using LR Clonase (Invitrogen). Similarly, the control Ad-GFP virus was generated. The coding sequence of the C2 domain of human PLCη2 (PLCη2_C2; amino acids 741–872) was amplified from the full-length human *Plch2* mRNA transcript (encoding amino acids 1–1,416) expressed in transfected cells using RT‒PCR. The Ad-PLCη2 and Ad-PLCη2_C2 vectors were constructed as described above. To amplify the recombinant adenoviruses, each plasmid was linearized with Pac I and was transfected into 293A cells using Lipofectamine 2000. The transfected 293A cells were cultured for 5–10 days in Dulbecco’s modified Eagle medium containing 10% FBS, and the medium was replaced every 2–3 days. Viruses from the culture supernatants of 293A cells that showed cytopathogenic effects were purified using an Adeno-X Purification Kit (Clontech Laboratories, 631533), titrated by performing plaque assays with a dilution series and stored at −80 °C until use.

For PLCη2 overexpression and knockdown, we used an OmicsLink open reading frame expression plasmid (pReceiver-PLCη2 expression vector; GeneCopoeia, EX-H0612-M61) and an OmicsLink short hairpin RNA (shRNA) clone (psi-H1-PLCη2 shRNA; GeneCopoei, HSH023033). The pEZ-M61 plasmid expressing eGFP and the psi-H1 plasmid expressing shRNA were used as the respective control vectors. The DNA and shRNA vectors were transfected separately into cells using the Human Dermal Fibroblast Nucleofector Kit (Lonza) according to the manufacturer’s protocol. In brief, 5 × 10^5^ cells were resuspended in 100 μl of nucleofector solution containing DNA or shRNA plasmids and electroporated with the U-23 program on an Amaxa Nucleofector II device.

### Screening for genes related to RA FLS proliferation and NF-κB activity using functional C2 domainomics-based HTS

To construct expression vectors encoding GFP-tagged C2 domains, we generated a C2 domain library using the sequences of 144 different C2 domains with the Gateway adenovirus system, as described above. Next, we performed functional C2 domainomics-based HTS to screen for candidates. The final candidates were classified and selected on the basis of the degree of their effects on RA FLS proliferation and NF-κB activation. RA FLS proliferation was assessed using Cell Counting Kit-8 (CCK-8) assays (Dojindo, CK04). To monitor NF-κB signaling activity, we performed secreted luciferase reporter assays, as described below.

### Cell proliferation and cell viability assays

Cell proliferation and cell viability assays were performed as previously described^8–12^. In brief, the assays were performed by seeding cells in either 96-well plates (2 × 10^3^ cells per well) or 24-well plates (2.0 × 10^4^ cells per well). On the following day, the test reagents were diluted in medium and added to the wells. Two or three days later, the medium was removed, and CCK-8 solution or MTT solution (Sigma-Aldrich, M5655) was added to each well according to the manufacturers’ instructions. The absorbance values were measured using a microplate reader (Molecular Devices). The relative absorbance values under each condition were calculated by dividing the signal intensities in the treated wells by those in mock-treated wells in three independent experiments.

### NF-κB activity assay

NF-κB activation was monitored using the Ready-to-Glow Secreted Luciferase Reporter Assay (Clontech, PT3902) and the NF-κB p65 Transcription Factor Assay Kit (Abcam, ab133112) according to the recommended experimental protocols. To perform the secreted luciferase reporter assays, we utilized RA FLSs harboring the pNFκB-MetLuc2 vector (Clontech). NF-κB activation was assessed in response to TNF (25 ng/ml) treatment after infection with each recombinant adenovirus from the library, which expressed a distinct C2 homolog. Cells harboring the pNFκB-MetLuc2 vector in 24-well plates (2 × 10^4^ cells per well) were infected separately with all 144 adenoviruses in the C2 library (100 MOI), and the supernatants were collected at 24 h post infection. The luciferase activity in each supernatant was measured using the Ready-to-Glow Secreted Luciferase Reporter Assay and the Promega GloMax-Multi Detection System.

NF-κB DNA-binding activities in PLCη2-overexpressing MH7A cells were determined 48 h after treatment with IL-1β and/or TG, at which time nuclear protein extracts were prepared using the NE-PER Nuclear and Cytoplasmic Extraction Reagents (Thermo Fisher, 78833). The extracted nuclear fractions were added to black 96-well plates containing the consensus binding sequence for NF-κB p65 and incubated for 1 h. Subsequently, a chicken reactive rabbit anti-NF-κB p65 antibody (Abcam, ab16502) was added to each well, and the plates were incubated for 1 h. After washing, an HRP-conjugated secondary goat anti-rabbit antibody (Invitrogen, 31460) was added to each well, and the plates were incubated for 1 h. Each well was washed five times, loaded with a developing solution and then incubated for 15–45 min. The optical density at 450 nm (OD_450_) of each well was measured using a VersaMax microplate reader (Molecular Devices, SoftMax Pro 6.5.1), after the addition of stop solution to each well. The OD_450_ values were determined in triplicate. The OD_450_ data were expressed as percentages of those found with control GFP-expressing cells.

### Assessing the in vivo invasion of RA FLSs into human cartilage implants

Mouse experiments were performed as described previously^8–12^. Four-week-old severe combined immunodeficiency (SCID) mice (Charles River) were housed under standardized conditions under specific pathogen-free conditions with water and food provided ad libitum. As previously described, the animals underwent inverse-wrap implantation^32,33^. In brief, on the day of implantation, normal human cartilage was obtained and cut into pieces. An incision was made in each cube of the insert sponge (80 mm^3^ sponge; Gelfoam, Pfizer), and a piece of cartilage (50 mm^3^) was inserted. One piece of cartilage, which was used as a control for scoring after implantation, was immediately snap-frozen and stored at −70 °C. Each sponge was soaked with 5 × 10^5^ cells suspended in sterile saline. These pieces of sponge containing cells and cartilage were inserted into the skin of anesthetized mice under sterile conditions. After 60 days, the implants were removed from the euthanized mice, immediately embedded, snap-frozen and stored at −70 °C until further use. The explants were stained with hematoxylin and eosin (H&E) using a standard protocol, and each specimen was examined for the grade of FLS invasion into the cartilage and erosion, as described previously^8,10–12,34^. Cells with similar characteristics (such as shape and size) could be counted using this system. Three measurements were made for each unit area, and the mean values were calculated. The outcomes were measured by blinded observers. All animals were randomly allocated to separate groups, and no animals were excluded from the study. The invasion of synovial tissue into cartilage tissue was quantified according to a semiquantitative score ranging from 0 to 4, which was based on the number of invading cell layers and the number of affected cartilage sites. Erosion was scored on a scale of 0 to 4, as described previously^33^.

### In vivo Matrigel plug assay and TUNEL assay

To assess FLS apoptosis in vivo, we performed in vivo Matrigel plug assays and terminal deoxynucleotidyl transferase dUTP nick-end labeling (TUNEL) assays and then stained the cells. RA FLSs (1 × 10^6^) were infected with an adenoviral vector encoding GFP, PLCη2 or PLCη2_C2 for 24 h and then mixed with 250 μl of Corning Matrigel Matrix. Matrigel containing RA FLSs was injected into the abdomen of athymic nude mice (Jackson Laboratory). After 7 days, the skin of each mouse was removed to expose the Matrigel plugs, which remained intact. After any quantitative differences were noted and photographed, the Matrigel plugs were fixed in 4% formalin, embedded in paraffin, sectioned, deparaffinized and stained (TUNEL, H&E and immunohistochemistry (IHC)). For TUNEL staining, we used the DeadEnd Colorimetric TUNEL system (Promega, G7130) following the manufacturer’s instructions. In brief, the slides were incubated in permeabilization solution (0.1% Triton X-100 in 0.1% sodium citrate buffer) for 2 min on ice and rinsed twice with phosphate-buffered saline, after which 50 µl of TUNEL reaction mixture was added. After a 30-min incubation at 37 °C, the substrate solution was added. After incubation for 10 min, the slides were mounted and examined under a light microscope. Quantitative data were obtained using microscopy by counting the number of positively stained cells per unit area in digitalized images covering the whole Matrigel area. H&E and IHC staining were performed as previously described^8–12^. Antigen retrieval was performed by autoclaving for 15 min. After the sections were incubated with blocking solution for 30 min, they were further incubated with anti-cPARP (1:1,000; Cell Signaling Technology, 5625) and anti-Bax (1:1,000; Santa Cruz Biotechnology, sc-7480) antibodies for 1 h, with a biotinylated secondary antibody for 20 min, and then with streptavidin–horseradish peroxidase for 10 min. Visualization was achieved with 3,3'-Diaminobenzidine (DAB) chromogen, and the sections were counterstained with hematoxylin.

### Ca^2+^ imaging, BiFC assays and co-IP

For Ca^2+^ imaging, bimolecular fluorescence complementation (BiFC) analysis and co-immunoprecipitation (co-IP), we constructed expression vectors encoding full-length human *Plch2* (NM_014638.3) and human *HRas* (NM_005343.4). Both full-length genes were amplified using RT‒PCR and cloned and inserted into several destination vectors (pDEST-mCherry-N, pDEST-VN-N, pDEST-VC-N, pDEST-GFP-N and pDEST-HA-N) using the Gateway Cloning System (Invitrogen).

For Ca^2+^ imaging, MH7A cells plated on glass coverslips were transfected with mCherry-control or mCherry-PLCη2_C2 expression vectors using Lipofectamine 2000 (Life Technologies). At 24 h posttransfection, the cells were incubated with 5 µM Fura-4 AM for 20 min at 37 °C and 5% CO_2_, after which the coverslips were transferred to an imaging chamber with normal HEPES buffer (135 mM NaCl, 5.4 mM KCl, 1 mM MgCl_2_, 1.8 mM CaCl_2_, 5 mM HEPES and 10 mM glucose, pH 7.3). mCherry and GFP fluorescence was monitored with a confocal laser-scanning microscope (Nikon A1) at 2 s intervals for 120 s. To stimulate Ca^2+^ levels, the HEPES buffer was replaced with 2 mM CaCl_2_ and 10 µM ionomycin. Ionomycin is a Ca^2+^ ionophore that initially releases calcium from the ER but later allows an influx of extracellular Ca^2+^.

For BiFC analysis, PLCη2, the PLCη2 C2 domain, the PLCη2 C2 deletion mutant and HRas proto-oncogene GTPase (HRAS) were cloned into the pBiFC-VN173 and pBiFC-VC155 vectors (Addgene). HEK293T cells were cotransfected with N-terminal-tagged BiFC vectors and the RFP control vector. The next day, the cells were fixed with 4% paraformaldehyde for 20 min at room temperature and mounted with Dako Fluorescence Mounting Medium. Venus fluorescence signals were observed by confocal microscopy (Nikon A1).

For co-IP in overexpressed HEK293T cells, whole-cell lysates were mixed overnight at 4 °C with 1 mg/ml anti-GFP (B-2; Santa Cruz Biotechnology) antibody in lysis buffer (5 mM HEPES pH 7.5, 250 mM NaCl, 0.5% NP-40, 10% glycerol and 2 mM EDTA) containing a protease-inhibitor cocktail (Roche). The immune complexes were incubated by binding to mixed protein A/G PLUS agarose (Santa Cruz Biotechnology) for 1 h and then washed with lysis buffer. For immunoblotting, protein samples were separated by SDS‒PAGE on 10% gels. The separated proteins were transferred to polyvinylidene fluoride membranes (Bio-Rad). The blots were incubated overnight at 4 °C with anti-HA (Roche Applied Science; 3F10, 1:1,000) and anti-GFP (Santa Cruz Biotechnology, B-2, 1:1,000) antibodies. The blots were washed and incubated with horseradish peroxidase-conjugated goat anti-rat (Jackson Laboratory, 1:3,000) or anti-mouse light chain (Jackson Laboratory, 1:3,000) antibodies, followed by washing and detection of immune reactivity using enhanced chemiluminescence (Bio-Rad).

### HRAS activity assay

Total proteins (500 μg) from whole-cell extracts were subjected to active-HRAS pulldown analysis (H-Ras Activation Assay Kit; Abcam, ab211158) according to the manufacturer’s protocol. In brief, for the RAS-GTP assays, a recombinant GST-tagged Raf1–Ras binding domain fusion protein was mixed with glutathione resin and cell lysates for 1 h at 4 °C. The samples were subsequently washed three times with lysis buffer and then released from the resin by adding SDS‒PAGE sample buffer. The proteins were separated by SDS‒PAGE and then analyzed by IB. The activated RAS isoforms were detected with matching antibodies. Total lysates (30 μg; the same amount used for the pulldown assays) were separated and subjected to IB analysis for HRAS, phospho-ERK1/2, Thr202/Tyr204 (1:1,000; Cell Signaling Technology, 9101) and total-ERK1/2 (1:1,000; Cell Signaling Technology, 9102).

### Phosphorylated protein arrays (Phospho Explorer Antibody Array)

The relative phosphorylation levels of the selected target molecules involved in signal transduction pathways in MH7A cells were analyzed using three types of commercially available Human Pathway Explorer Phosphorylation Antibody Array C1 kit (RayBiotech). The Human NF-κB Pathway Phosphorylation Array C1 (RayBiotech, AAH-NFKB-1-8) was designed for parallel detection of 11 downstream signaling molecules, the Human JAK/STAT Pathway Phosphorylation Array C1 (RayBiotech, AAH-JAKSTAT-1-8) was designed for parallel detection of 12 downstream signaling molecules, and the Human/Mouse MAPK Phosphorylation Array (RayBiotech, AAH-MAPK-1-8) was designed for parallel detection of 17 downstream signaling molecules. Whole-cell lysates from cultured MH7A cells expressing GFP or PLCη2 were treated for 1 h with IL-1β and TG and collected using protein extraction buffer (Full Moon BioSystems). After protein quantification using the Bradford method, 1 mg of each protein sample was loaded onto a RayBio Human Signaling Pathway Antibody Array membrane. After incubation with horseradish peroxidase-conjugated anti-rabbit IgG, a chemiluminescence imaging system (ChemiDoc Imaging System, Bio-Rad) was used to analyze the results. The internal control signals of each protein array chip were used for standardization purposes. The signal intensities were determined using densitometric analysis using Bio-Rad Image Lab software (version 6.0).

### CIA and intraarticular injection

Collagen-induced arthritis (CIA) was induced as described previously^8–12^. In brief, the mice were divided into the following five groups: (1) Ad-GFP plus CIA, (2) Ad-PLCη2 plus CIA and (3) Ad- PLCη2_C2 plus CIA (*n* = 10–15 mice per group). A single intraarticular injection of 10 μl of adenovirus (3 × 10^9^ plaque-forming units (PFU)) or phosphate-buffered saline was administered to each ankle joint on day 20. Disease severity was scored by visual inspection of the paws, using a scale of 0–4 for each paw (0, no swelling; 1, slight swelling and erythema; 2, moderate swelling and erythema; 3, severe swelling and erythema; 4, maximal inflammation with joint stiffness). The maximum total score was 16. Scoring was performed by two independent observers in a blinded manner. On day 35, the diameter of each ankle was measured using a digital microcaliper placed across the ankle joint at the widest point. The mice were then euthanized, and the joint tissues were collected from each animal for end-point histology to evaluate the effects of adenovirus injection. For histopathological analyses, mouse ankle joint tissues were fixed overnight with 10% formalin, decalcified for 3 weeks in 10% EDTA and dehydrated. The tissues were subsequently embedded in paraffin. Serial 5-μm-thick sections were prepared using a microtome. The ankle joint tissue sections were stained with H&E and safranin O and subjected to tartrate-resistant acid phosphatase (TRAP) staining. The stained ankle-joint sections were scored using a semiquantitative scoring system (0–5 scale) according to synovial inflammation, cartilage damage and bone erosion.

### Statistical analysis

All experiments were independently repeated at least three times, with normalization applied to minimize baseline variability. The normality of the data distribution was verified using the Kolmogorov‒Smirnov test or Shapiro‒Wilk test. Two-tailed, unpaired Student’s *t*-test, the Mann‒Whitney *U* test or the Wilcoxon rank-sum test was used for comparisons; otherwise, one-way analysis of variance (ANOVA) with Tukey’s multiple comparisons or the Kruskal‒Wallis, Brown‒Forsythe or Welch’s ANOVA test was used for comparisons among multiple groups. Statistical significance was set at *P* < 0.05. Analysis was performed using GraphPad Prism (version 6), Excel and Minitab 14.0 statistical software.

### Study approval

All animal experimental procedures and protocols were approved by the Institutional Animal Care and Use Committee of Gyeongsang National University (GNU-160603-M0028) and were performed in accordance with the Guide for the Care and Use of Laboratory Animals prepared by the National Academy of Sciences. Procedures involving specimens obtained from human subjects were performed according to a protocol approved (2014-02-013) by the institutional review board of Gyeongsang National University Hospital.

## Results

### Functional C2 domainomics-based HTS identifies the C2 domain of PLCη2 as a regulator of RA FLSs

**Fig. 1:**
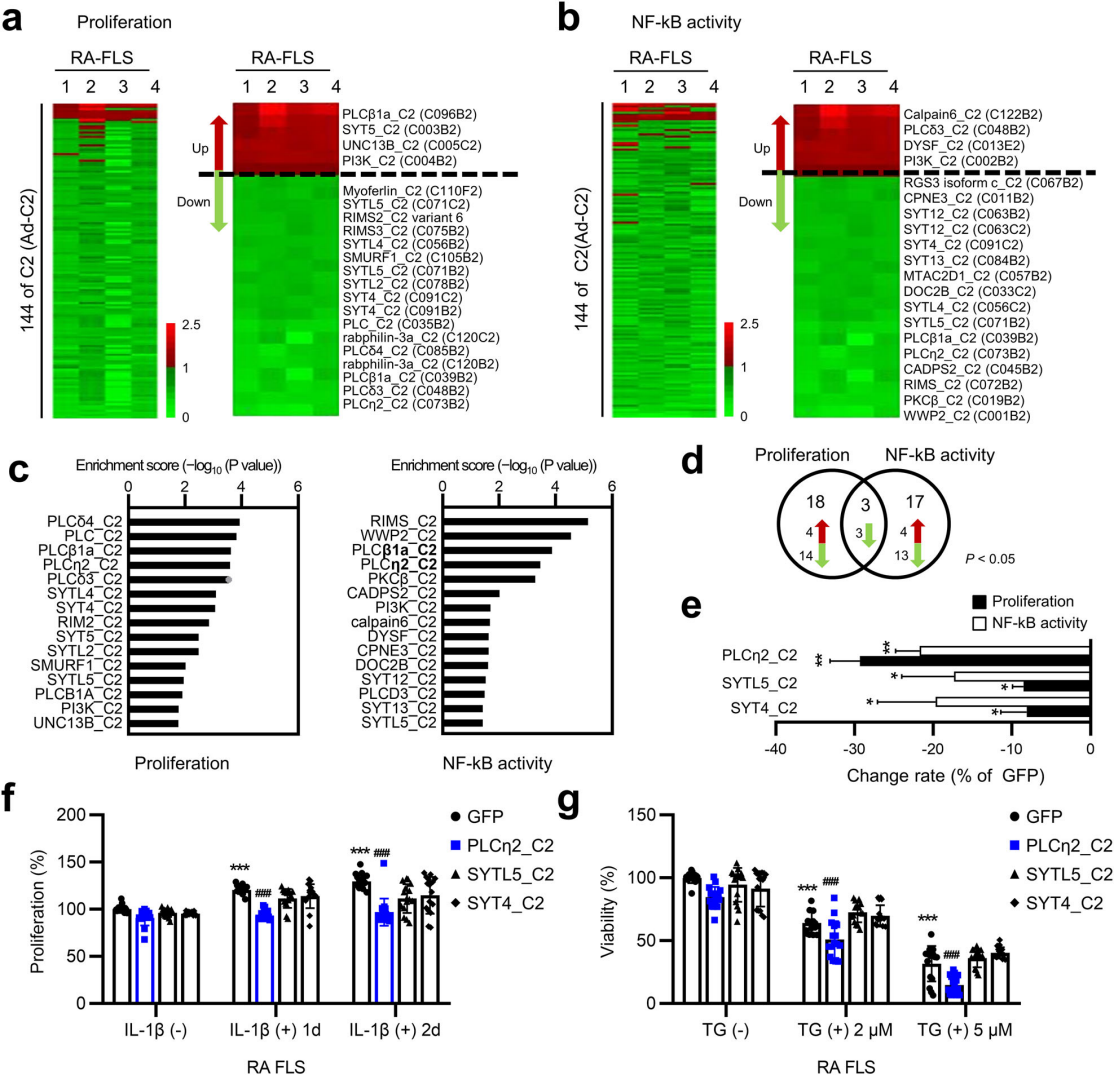
Screening process and selection of the PLCη2_C2 domain, which affects the proliferation and NF-κB activity of RA FLSs. **a**, **b** Cell proliferation rates (**a**) and relative NF-κB activities (**b**) were measured in RA FLSs transduced separately with 144 different Ad-C2 vectors or the Ad-GFP vector for 48 h. Heatmaps display differences in the cell proliferation rate (**a**, left) and relative NF-κB activity (**b**, left). The heatmap areas show that Ad-C2 significantly altered the cell proliferation rate (**a**, right) and relative NF-κB activity (**b**, right). Red and green shading represents increased or decreased cell proliferation rates and NF-κB activities in the transduced RA FLS, respectively, compared with the corresponding values in the control transductants (Ad-GFP). **c** Top 15 Ad-C2 vectors in terms of altered cell proliferation rates (left) and relative NF-κB activities (right). **d** Venn diagrams showing the numbers of Ad-C2 vectors associated with significantly altered cell proliferation rates and relative NF-κB activities. **e** The overlap between Ad-C2 vectors associated with altered cell proliferation rates and altered NF-κB activities. The data shown represent vectors that led to reduced cell proliferation rates and NF-κB activities in RA FLSs at the same time compared with those obtained with Ad-GFP. Only Ad-C2 vectors with an enrichment score (−log(*P* value)) above 1.3 (*P* < 0.05) were considered for the analysis. *P < 0.05, **P < 0.01, versus the vector control (Ad-GFP). **f**, **g** Cell proliferation rates (**f**) and viabilities (**g**) of RA FLSs transduced with Ad-GFP, Ad-PLCη2_C2, Ad-SYTL-C2 or Ad-SYT4_C2 for 48 h. The data shown are expressed relative to vector control (Ad-GFP) values and represent the mean ± s.e.m. of three independent experiments involving three different RA patients. *P < 0.05, **P < 0.01, ***P < 0.001 versus control (untreated); ^###^*P* < 0.001 versus vector control (Ad-GFP), as determined by unpaired Student’s *t*-test (**e**) and one-way ANOVA followed by unpaired, two-tailed *t*-test (**f** and **g**).

Using an HTS-based approach to screen for and verify the tumor-like behaviors of RA FLSs, we designed an adenoviral cDNA expression library targeting 144 different C2 domains, which were selected with the Pfam database^26^. A screen was conducted after both IL-1β and TNF treatment to address the effects of IL-1β-induced proliferation and TNF-stimulated NF-κB activation in RA FLSs. In our initial filtering steps, candidate C2 domains that dysregulated the RA FLSs were identified by applying both significance and fold-change thresholds, resulting in the selection of 21 candidates from the proliferation screen (Fig. 1a) and 20 candidates from the NF-κB activity screen (Fig. 1b). Figure 1c shows the top 15 Differentially Expressed Genes (DEGs) when the C2 domains were ranked according to the absolute fold-change differences in terms of IL-1β-induced proliferation and TNF-stimulated NF-κB activation. We identified three C2 domain-expressing adenoviruses (Ad-C2) as positive candidates for further study (Fig. 1d, e).

Because proliferation assays reflect only viable cell numbers and do not distinguish contributions to cell death, we assessed the effects of all three Ad-C2 vectors on TG-induced apoptosis. We confirmed that IL-1β-induced proliferation and TG-induced cell death were reduced by the C2 domains of PLCη2 (PLCη2_C2), synaptotagmin-like protein 5 (SYTL5_C2) and synaptotagmin 4 (SYT4_C2) (Fig. 1f, g). Among these three candidates, we investigated the effect of PLCη2_C2, which had the most profound effect on proliferation and NF-κB activity in the human RA FLS cell line MH7A, suggesting that PLCη2_C2 may be important in regulating RA FLS pathogenesis.

### PLCη2_C2 represses hyperproliferation and an aggressive phenotype in primary RA FLSs

**Fig. 2:**
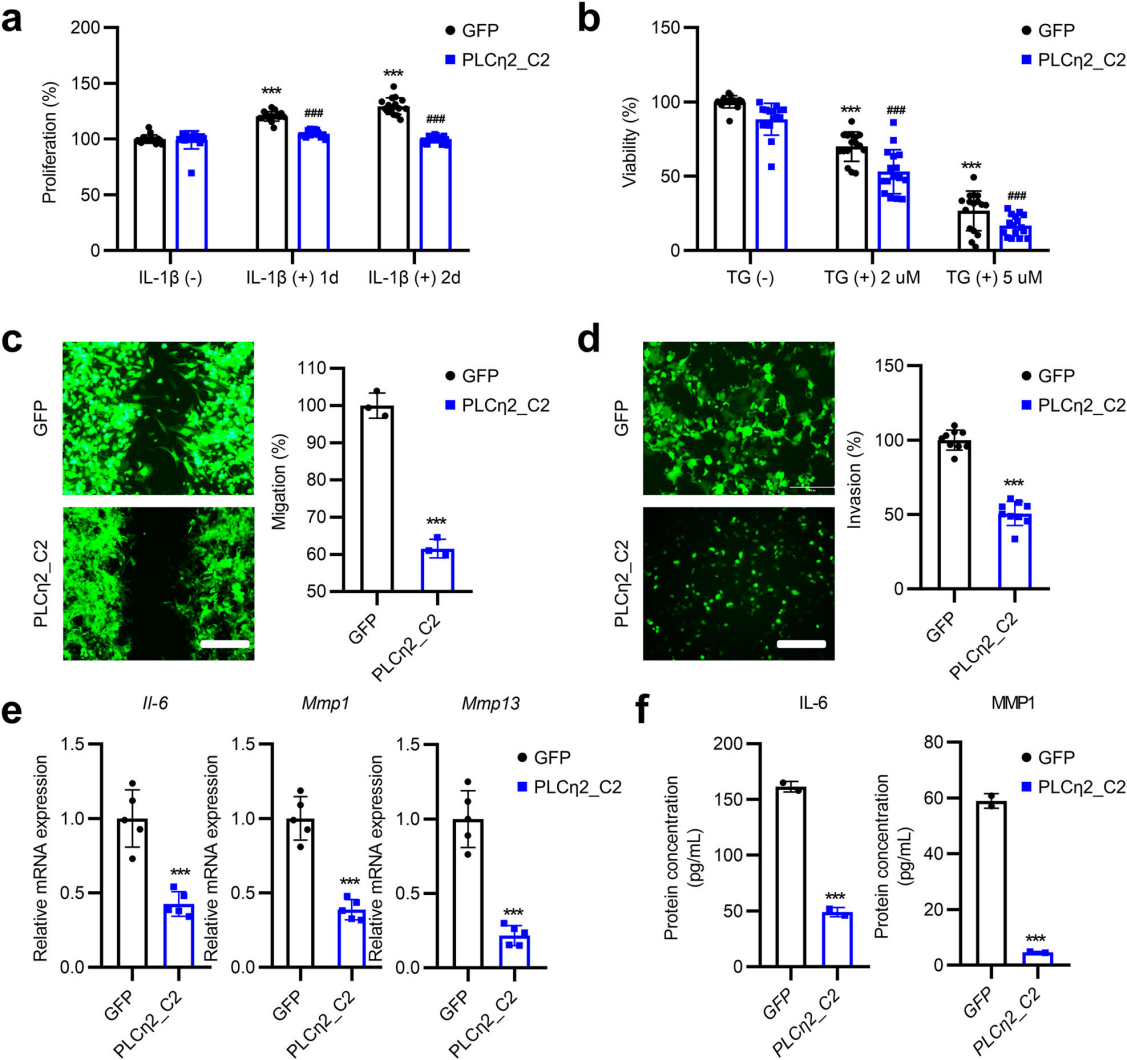
Effects of the PLCη2_C2 domain on the aggressive and inflammatory phenotypes of RA FLSs. **a**, **b** Ad-GFP- and Ad-PLCη2_C2-infected RA FLSs were treated with IL-1β (**a**) and/or 2 or 5 μM TG (**b**). The cell proliferation rates (**a**) and/or viabilities (**b**) were subsequently analyzed using MTT and CCK-8 assays. The results were calculated as percentages relative to the data obtained from untreated Ad-GFP-infected RA FLSs. The graphs in **a** and **b** present the mean ± s.e.m. of three independent experiments. **c**, **d** The effect of PLCη2_C2 overexpression on the migration and invasion of RA FLSs. Representative images are shown (original magnification, ×40). The relative migration (**c**) and invasion (**d**) data shown represent the mean ± s.e.m. of four independent experiments involving samples from three different patients with RA. **e** Determination of *Il-6*, *Mmp1* and *Mmp13* mRNA expression levels by qRT‒PCR in PLCη2_C2-overexpressing RA FLSs. The data shown are presented as the mean ± s.e.m. **f** Quantification of IL-6 and MMP1 serum levels in PLCη2_C2-overexpressing RA FLSs. ***P < 0.001 versus control (untreated); ^###^*P* < 0.001 versus vector control (Ad-GFP) as determined by one-way ANOVA followed by unpaired, two-tailed *t*-test (**a** and **b**) or unpaired Student’s *t*-test (**c**–**f**).

To explore the potential role of PLCη2_C2 in RA, we monitored the IL-1β-induced proliferation of primary RA FLSs with adenovirus-mediated PLCη2_C2 overexpression. PLCη2_C2 overexpression reduced RA FLS proliferation compared with the proliferation of GFP vector control cells (Fig. 2a). Given that RA FLS hyperplasia depends on apoptosis resistance^5^, we examined whether PLCη2_C2 could regulate apoptosis. To do this, we stimulated RA FLSs with TG for 72 h. The viability of PLCη2_C2-overexpressing RA FLSs in the presence of TG was significantly lower than that of GFP vector control cells (Fig. 2b), and 3-(4,5-Dimethylthiazol-2-yl)-2,5-Diphenyltetrazolium Bromide (MTT) assays revealed that PLCη2_C2 overexpression increased cell death (Fig. 2b). These data suggest that PLCη2_C2 regulates RA FLS proliferation and death and that, with enhanced PLCη2 activity and/or expression, RA FLSs are more susceptible to apoptosis.

During inflammation, RA FLSs undergo phenotypic changes and acquire an aggressive phenotype^5,7^. Furthermore, their destructive behavior involves essential steps that lead to joint destruction in individuals with RA^1^. Therefore, we assessed the effect of PLCη2_C2 on the aggressive phenotypes of RA FLSs. To assess the effect of PLCη2_C2 on direct migration, we performed in vitro scratch-wound healing assays. The results revealed reduced migration of IL-1β-treated Ad-PLCη2_C2-transduced RA FLSs (Fig. 2c). The aggressive destruction of cartilage and bone tissues is a key pathogenic behavior of RA FLSs; the in vitro invasion potential of RA FLSs is strongly associated with the rate of joint destruction in patients with RA^1,2^. Therefore, we determined the effect of PLCη2_C2 overexpression on modulating RA FLS invasion by conducting Transwell assays with Matrigel-coated membranes. RA FLSs overexpressing PLCη2_C2 presented lower invasion than the GFP vector controls (Fig. 2d). Collectively, our findings indicate that PLCη2_C2 overexpression impaired both the migratory and invasive abilities of RA FLSs.

Cytokines produced by RA FLSs play important roles in joint inflammation and damage. Thus, we conducted quantitative reverse transcription‒PCR (qRT‒PCR) and enzyme-linked immunosorbent assay experiments, which revealed that PLCη2_C2 overexpression reduced the expression and secretion of several proinflammatory cytokines (for example, IL-6) and Matrix Metalloproteinases (MMPs) in RA FLSs (Fig. 2e, f). Taken together, our data suggest that the C2 domain of PLCη2 modulates the transformation of FLSs toward more aggressive phenotypes during RA.

### PLCη2 downregulation in the synovial tissues and FLSs of patients with RA

**Fig. 3:**
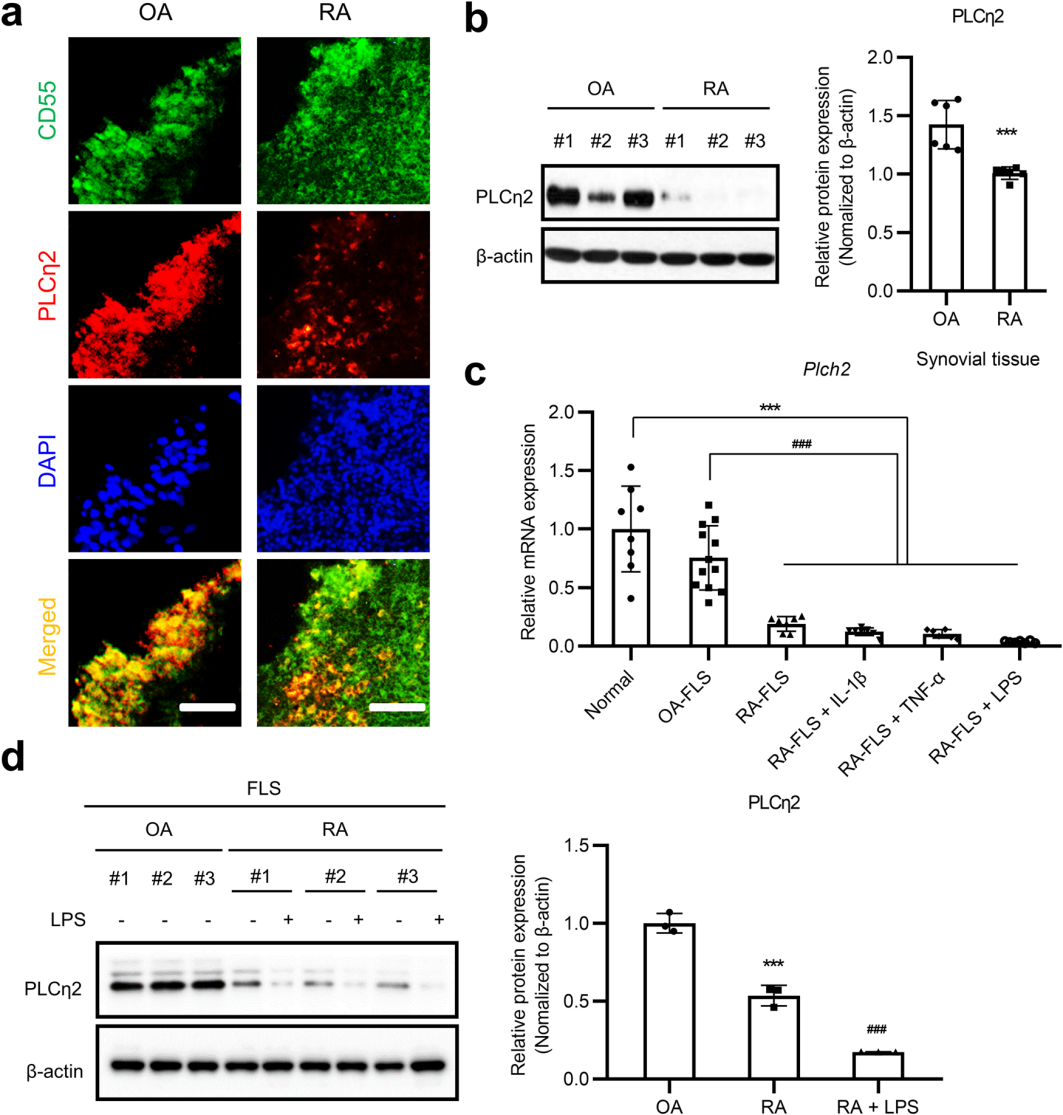
Comparison of PLCη2 expression between RA and OA joint tissues and FLSs. **a** Representative immunofluorescence (IF) images of synovial tissues from patients with OA or RA. The IF images show costaining for PLCη2 (red) and CD55 (green). The nuclei were stained blue with DAPI. Scale bars, 50 μm. **b** PLCη2 expression in OA and RA synovial tissues was evaluated using IB analysis. The graph shows the quantification of the IB intensity for samples from three different patients with RA. ***P < 0.001 versus OA FLSs, as determined by the Mann‒Whitney *U* test. **c**, OA FLSs and RA FLSs were incubated in the absence (RA FLSs) or presence of 10 ng/ml IL-1β, TNF or LPS for 1 day, after which the *Plch2* mRNA expression in the cells was analyzed using RT‒qPCR. ***P < 0.001 versus normal tissues, ^###^*P* < 0.001 versus OA FLSs, based on one-way ANOVA with Bonferroni correction for multiple comparisons. **d** OA FLSs and RA FLSs were incubated in the absence or presence of 10 μg/ml LPS, and the cells were analyzed for PLCη2 protein expression using IB analysis. The graph shows the quantification of the staining intensity for samples from three different patients with RA. ***P < 0.001 versus OA FLSs; ^###^*P* < 0.001 versus untreated RA FLSs. Comparisons of numerical data between groups were performed using the unpaired *t*-test, Welch’s *t*-test, the Mann‒Whitney *U* test or Tukey’s multiple-comparisons test.

To test potential associations between PLCη2 and its C2 domain and real-world RA pathology, we first measured PLCη2 protein expression levels in synovial tissues and FLSs obtained from patients with RA or osteoarthritis (OA). PLCη2 protein expression was significantly downregulated in RA synovial tissues and FLSs (Fig. 3a, b) compared with the corresponding levels in the OA group, indicating that PLCη2 may play a role in RA synovial tissues and FLSs.

To imitate the inflammatory microenvironment in the joint cavities of patients with RA^5^, we stimulated RA FLSs with IL-1β, TNF and LPS. *Plch2* mRNA expression decreased during stimulation with IL-1β, TNF or LPS (Fig. 3c). LPS reduced PLCη2 protein expression to a greater extent in RA FLSs than in OA FLSs (Fig. 3d).

### PLCη2 negatively regulates hyperproliferation and an aggressive phenotype of RA FLSs

**Fig. 4:**
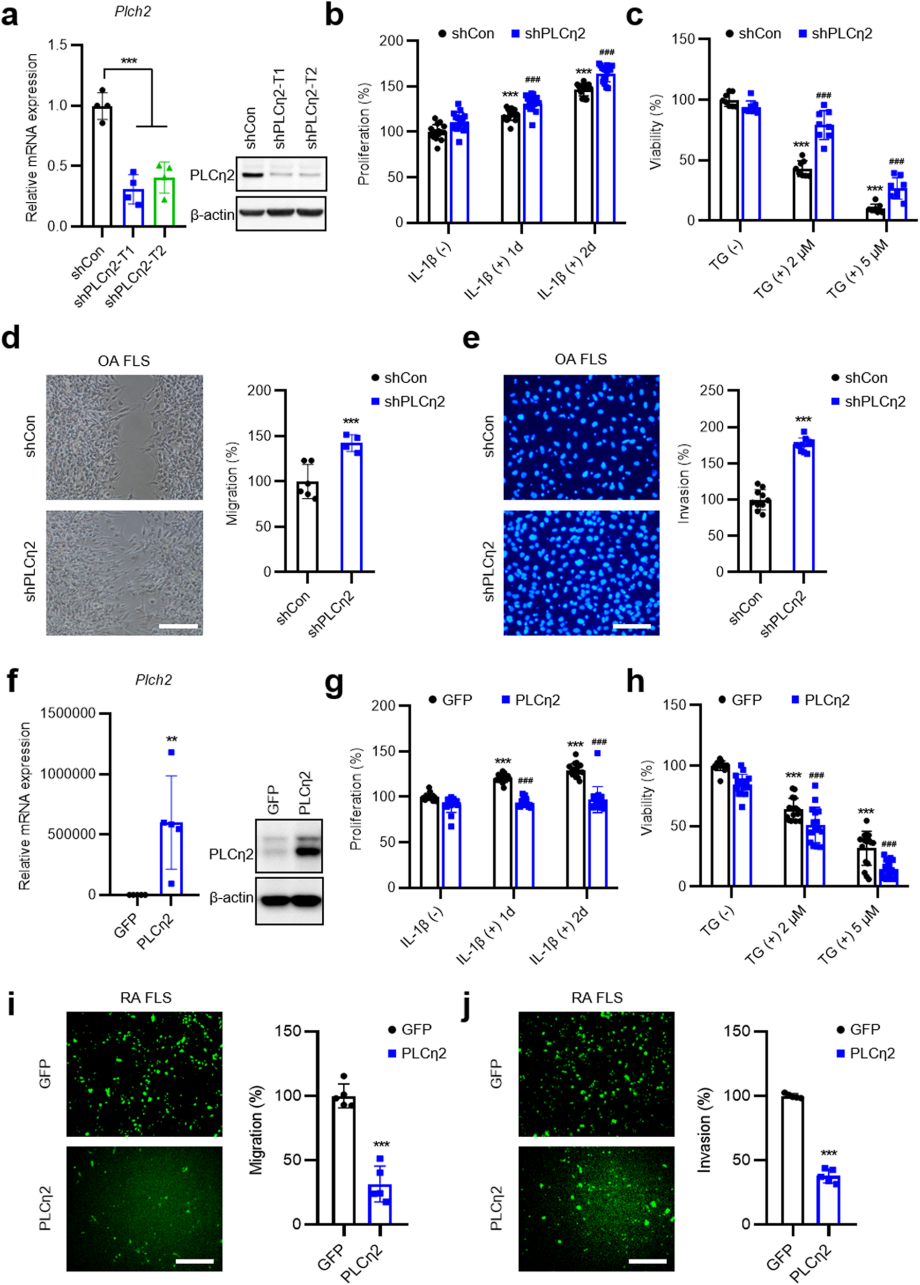
Effects of full-length PLCη2 on the aggressiveness of RA FLSs and OA FLSs. **a** mRNA and protein expression levels of PLCη2 in OA FLSs transfected with shPLCη2. To determine the activity of the PLCη2 shRNA, we used two independent PLCη2-specific shRNAs (target 1 (T1) and target 2 (T2)) as described in the Methods. A control, scrambled shRNA (shCon), was used as a negative control. **b**, **c** The effect of PLCη2 knockdown on the proliferation (**b**) and viability (**c**) of OA FLSs. The graphs indicate the relative proliferation and viability rates. **d**, **e** The effect of PLCη2 kno.ckdown on the migration (**d**) and invasion (**e**) of OA FLSs. Representative images are shown (original magnification, ×40). The relative migration and invasion data shown represent the mean ± s.e.m. of three independent experiments involving samples from three different patients with OA. **f** mRNA and protein expression levels of PLCη2 in RA FLSs infected with Ad-PLCη2. **g**, **h** The effect of PLCη2 overexpression on the proliferation (**g**) and viability (**h**) of RA FLSs. The graphs indicate the relative proliferation and viability rates. **i**, **j** The effect of PLCη2 overexpression on the migration (**i**) and invasion (**j**) of RA FLSs. Representative images are shown (original magnification, ×40). The relative migration and invasion data shown represent the mean ± s.e.m. of three independent experiments involving samples from three different patients with RA. In **a**–**j** **P < 0.01; ***P < 0.001, versus control (untreated, vector control); ^###^*P* < 0.001 versus vector control (shCon and Ad-GFP). Comparisons of numerical data between groups were performed using unpaired *t*-test, Welch’s *t*-test, the Mann‒Whitney *U* test or Tukey’s multiple-comparisons test.

We speculated that PLCη2 might modulate the tumor-like transformation and aggressive phenotype of FLSs; thus, we investigated the contribution of PLCη2 to proliferation and apoptosis by transfecting OA FLSs with an shRNA against *Plch2* transcripts (shPLCη2) or infecting RA FLSs with Ad-PLCη2. We found that shPLCη2 successfully downregulated PLCη2 in primary OA FLSs (Fig. 4a). Compared with the control shRNA-transfected plants, the shPLCη2 OA FLS-transfected plants exhibited increased proliferation (Fig. 4b) and decreased cell death (Fig. 4c). We also found that PLCη2 knockdown resulted in markedly increased cell migration and invasion (Fig. 4d, e).

By contrast, Ad-PLCη2-infected RA FLSs presented significantly higher PLCη2 mRNA and protein expression levels than did Ad-GFP-infected RA FLSs (Fig. 4f). As shown in Fig. 4g, IL-1β-induced RA FLS proliferation decreased after PLCη2 overexpression. In addition, PLCη2 overexpression significantly promoted TG-induced apoptosis in RA FLSs (Fig. 4h). Compared with Ad-GFP-infected control cells, RA FLSs overexpressing PLCη2 presented lower migration (Fig. 4i) and invasion of Matrigel-coated Transwell membranes (Fig. 4j). To rule out the influence of RA FLS heterogeneity in different patients^6^, we used MH7A RA FLSs to evaluate the effect of PLCη2 overexpression on FLS transformation. The same results were obtained with MH7A cells (Supplementary Fig. 1). Therefore, these data suggest that PLCη2 promotes apoptosis in RA FLSs and negatively regulates RA FLS migration and invasion.

**Fig. 5:**
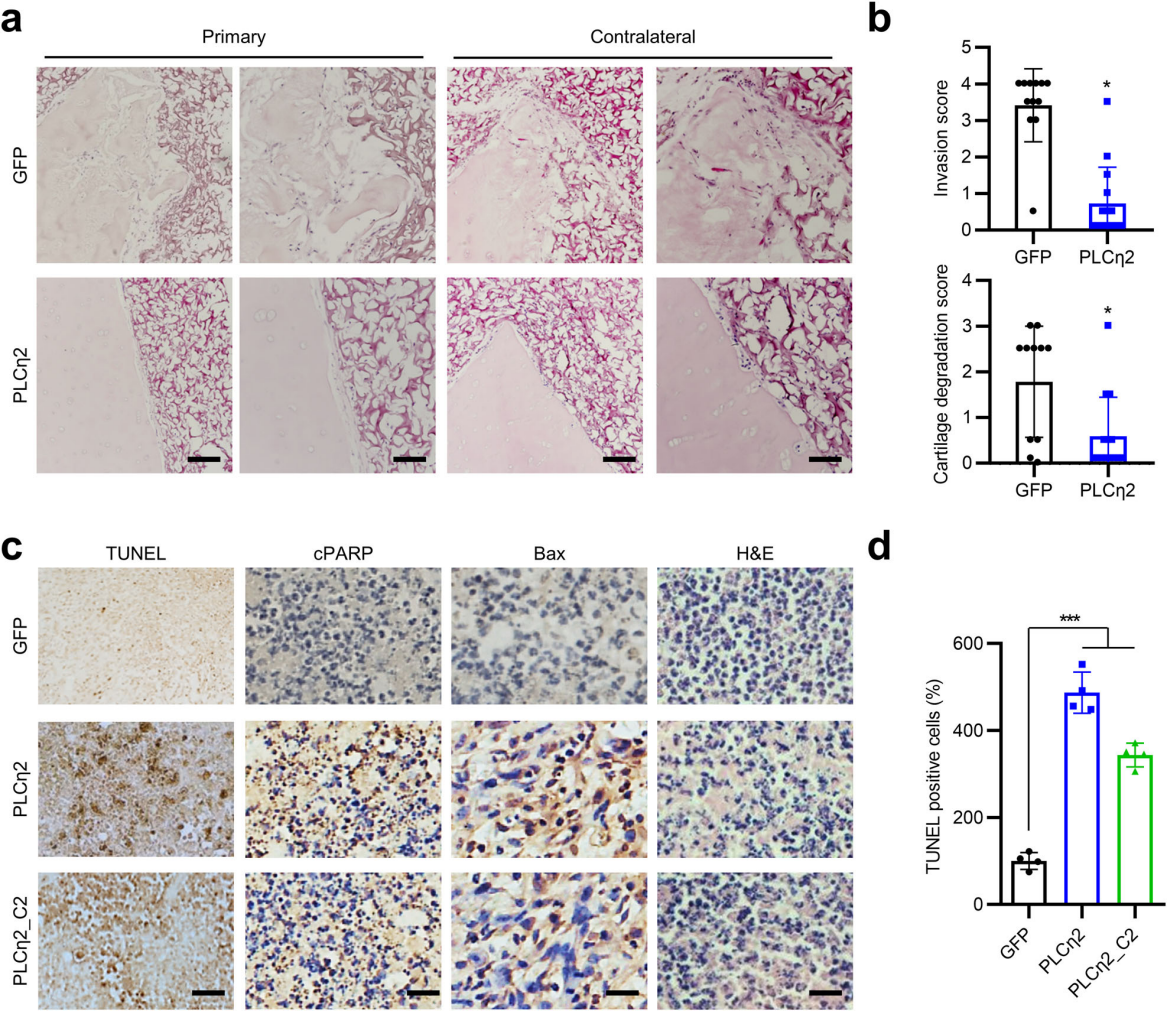
Inhibitory effects of PLCη2 and PLCη2_C2 against aggressive phenotypes in RA in an experimental mouse model. **a**, **b** The effect of PLCη2 overexpression on the invasion of RA FLSs into human cartilage implants administered subdermally to SCID mice: representative images of RA FLS invasion into cartilage (**a**; original magnification, ×100 (left); ×200 (right)); The graph indicating the invasion and erosion scores (**b**). The data shown represent the mean ± s.d. of three independent experiments *P < 0.05 versus the vector control (Ad-GFP), as determined by the Mann‒Whitney *U* test. **c**, **d** Effects of PLCη2 and PLCη2_C2 on RA FLS apoptosis in vivo. RA FLSs were implanted in Matrigel containing Ad-GFP, PLCη2, or PLCη2_C2, as described in the Methods. Representative H&E, TUNEL and IHC images of Matrigel-embedded RA FLSs from immunodeficient mice are shown. On day 7, the Matrigel plugs were excised for H&E, TUNEL and IHC staining for proapoptotic molecules (cPARP and Bax) (**c**). The data shown are expressed as a percentage of TUNEL-positive cells (**d**). The cells were manually counted at 200× magnification. The data shown are representative of three independent experiments. ***P < 0.001 versus Ad-GFP, as determined by the Kruskal‒Wallis test with Dunn’s multiple-comparisons test.

Finally, we determined the effects of PLCη2 and PLCη2_C2 on the in vivo aggressiveness of RA FLSs and their resistance to apoptosis. To evaluate the in vivo effect of PLCη2 overexpression on RA FLS invasion into cartilage, we used a SCID mouse coimplantation model, which lacks allogeneic immune responses and provides an ideal environment for studying interactions among human tissue components^32,33^. We confirmed that PLCη2 inhibited the ability of FLSs to damage cartilage in vivo. PLCη2-expressing recombinant adenovirus administration markedly decreased RA FLS invasion of cartilage in this model (Fig. 5a, b). The number of invasive cells in the PLCη2 group was significantly lower (*P* < 0.05) than in the vector control group. We also confirmed the effects of PLCη2 on RA FLS apoptosis in vivo using Matrigel plug implantation. Matrigel plugs containing Ad-PLCη2 or Ad-PLCη2_C2 showed significantly more apoptotic RA FLSs in vivo than the Ad-GFP control group (Fig. 5c, d). In addition, PLCη2 and PLCη2_C2 overexpression using recombinant adenovirus infection increased the expression of the proapoptotic proteins Bax and cleaved poly (ADP-ribose) polymerase (cPARP) (Fig. 5c). Taken together, these results show that PLCη2 and PLCη2_C negatively regulate the inflammation, proliferation, migration, invasion and apoptotic resistance of RA FLSs both in vitro and in vivo.

### PLCη2 and PLCη2_C2 drive the pathological signaling of RA FLSs in a Ca^2+^-independent manner

Many C2 domains function in a Ca^2+^-dependent membrane-binding manner and, hence, act as intracellular Ca^2+^ sensors^16,17^. For example, the C2 domains of PKC^35^ and synaptotagmin 1 (SYT1)^36^ induce Ca^2+^-dependent translocation to the plasma membrane. Because Ca^2+^-mediated PLCη2 signaling has not yet been reported, we investigated whether the C2 domain of PLCη2 would undergo a change in cellular localization in response to intracellular Ca^2+^. To monitor the dynamic processes of Ca^2+^ signaling and PLCη2_C2 translocation within the same cell, we examined the subcellular distribution of PLCη2_C2 after cytosolic Ca^2+^ alterations in transfected MH7A cells using ionomycin. We expressed a recombinant mCherry-PLCη2_C2 protein and the mCherry-control protein in RA FLSs treated with or without ionomycin and observed that both proteins were similarly expressed throughout the cells (except in the nucleus) after the Ca^2+^ increase (Supplementary Fig. 2). These results suggest that PLCη2_C2 associates with the plasma membrane in a Ca^2+^-independent fashion.

PLC activates the IP_3_ receptor (IP_3_R) on the ER, leading to emptying of the free Ca^2+^ pool, after which the influx of extracellular Ca^2+^ induces store-operated calcium entry (SOCE)^37,38^. We removed extracellular Ca^2+^ from the bath solution to determine whether an external or internal Ca^2+^ source was responsible for the PLC-induced Ca^2+^ change. TG, a specific ER Ca^2+^-ATPase inhibitor, is used as a pharmacological tool to deplete the internal Ca^2+^ store and bypass the receptors and subsequent signaling cascade that activates SOCE^37,38^. MH7A cells that were stably transfected with a mock or PLCη2 expression vector were labeled with the intracellular Ca^2+^ indicator Fura-2 AM and then stimulated with TG. The increase in the ratio of fluorescence emitted at 340 and 380 nm (F340/F380) was recorded to monitor the intracellular Ca^2+^ concentration. As shown in Supplementary Fig. 3, the Ca^2+^ concentration increased after TG-mediated stimulation. In the absence of extracellular Ca^2+^, TG elicited an initial increase in cytosolic Ca^2+^ due to passive release from ER Ca^2+^ stores (Supplementary Fig. 3). The intensity of the Ca^2+^ increase was similar for both stable transfectants, and after peaking at approximately 2 min after TG stimulation (approximately 100 s from the start of the experiment), the Ca^2+^ concentration began to decline slowly, and this decline was similar for both transfectants. The subsequent addition of extracellular Ca^2+^ enables a second phase of increased cytosolic Ca^2+^ concentrations resulting from store-operated calcium channel activation, as described for many cell types^39,40^. Inducing PLCη2 expression results in a doubling of store-operated calcium channel-mediated Ca^2+^ entry but does not affect Ca^2+^ release, which is consistent with the known properties of SOCE in many cell types^39–41^. An almost identical increase in SOCE activity was observed in the mock vector control group (Supplementary Fig. 3), indicating that PLC activity was not required for TG-induced, IP_3_R-mediated SOCE, at least with RA FLS pathogenesis. Taken together, these data indicate that the activity of PLCη2, at least in RA FLSs, functions in a Ca^2+^-independent manner.

### PLCη2 and PLCη2_C2 interact with HRAS

**Fig. 6:**
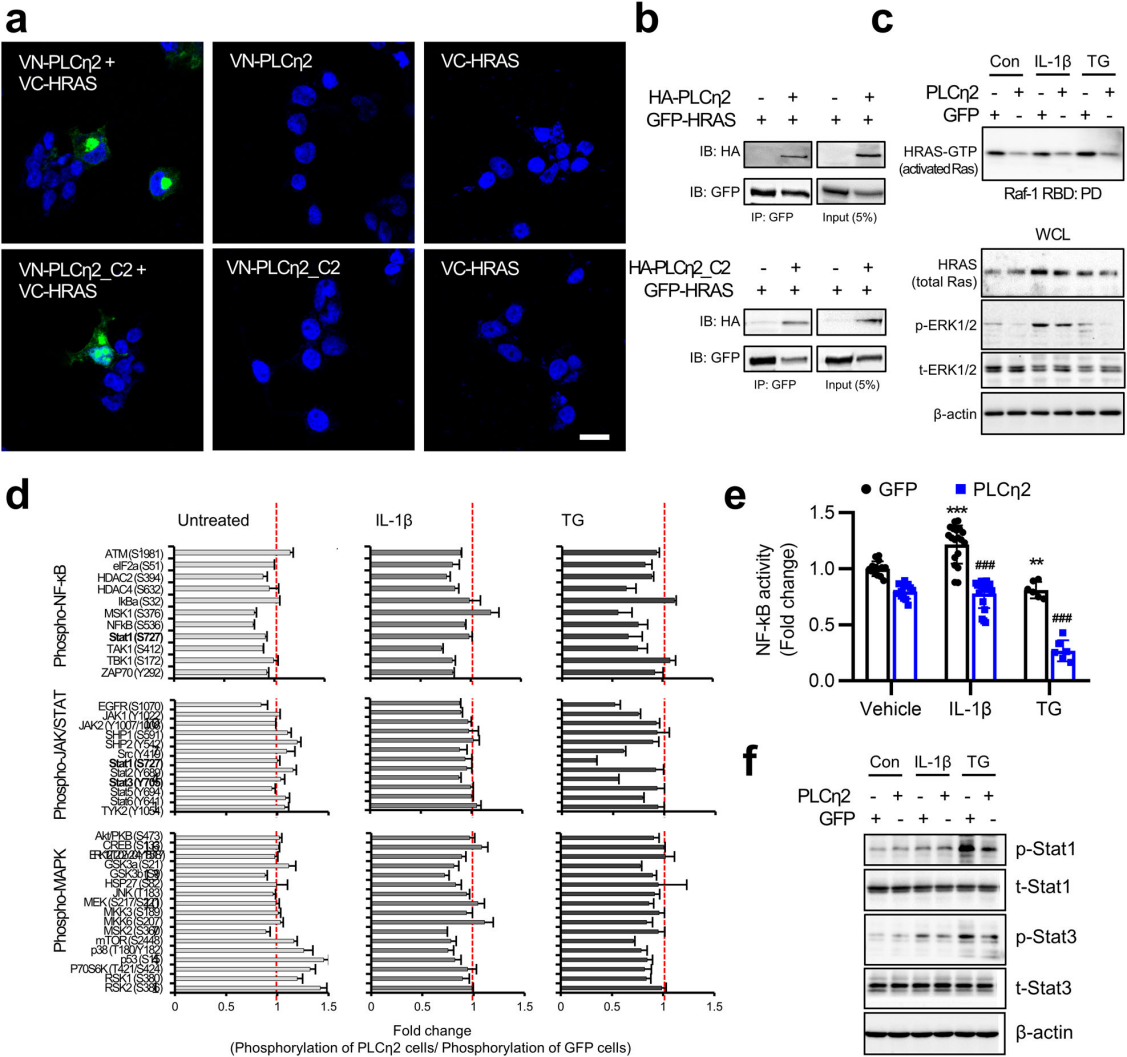
Interaction and effects of PLCη2 and PLCη2_C2 on HRAS and downstream signaling molecules in RA FLSs. **a** BiFC assays were performed in MH7A cells cotransfected with a VC-HRAS plasmid and a VN-PLCη2 (top) or VN-PLCη2_C2 plasmid (bottom). VN and VC are the N-terminal and C-terminal fragments of the Venus protein, respectively. The green fluorescence signals provide evidence of close proximity between the two proteins and indicate that VN-PLCη2 strongly interacts with HRAS-VC. Nuclei were visualized with DAPI (blue). Scale bars, 20 μm. **b** MH7A cells were transfected with HA–PLCη2, HA–PLCη2_C2 or GFP–HRAS plasmids, after which the resulting cell lysates were processed using IP using an anti-GFP antibody. After IP, the samples were subjected to IB analysis using an anti-HA or anti-GFP antibody. The input represents 5% of the cell lysate used in the co-IP assays. The co-IP data show that HA–PLCη2 bound to GFP–HRAS (top) and that HA–PLCη2_C2 interacted with GFP–HRAS (bottom). **c** The effect of PLCη2 overexpression on HRAS activity in MH7A cells. MH7A cells transfected with a GFP or PLCη2 plasmid were treated with 10 ng/ml IL-1β or 5 μM TG. HRAS activation (HRAS-GTP) was measured as described in the Methods. **d** Phosphoproteome array data for IL-1β- or TG-treated MH7A cells (NF-κB, JAK/STAT and MAPK signaling). A Phospho Explorer Antibody Array (described in the Methods) was used to screen total cell lysates from untreated, IL-1β-treated and TG-treated cells. The data show the fold changes in the expression levels of the indicated phosphoproteins upon IL-1β or TG treatment, with normalization to the results obtained with the GFP-expressing cells. The dotted line represents a fold change of 1 (no change) versus phosphorylation in the GFP-transfected MH7A cells. **e**, **f** Confirmatory NF-κB activity assays (**e**) and IB analyses (**f**) of GFP- and PLCη2-transfected MH7A cells treated with IL-1β or TG. **P < 0.01; ***P < 0.001 versus the control (vehicle); ^###^*P* < 0.001 versus the vector control (Ad-GFP) according to Brown–Forsythe and Welch’s ANOVA.

Many C2 domains are known to function in various signaling pathways through protein–protein interactions^16,17,42^. Thus, we searched for potential binding partners with PLCη2 using the BioGRID database (https://thebiogrid.org/), a repository of available physical and genetic interactions determined using various low- and high-throughput experimental assays and compiled through comprehensive curation. We found that HRAS may interact with PLCη2. Previous data have shown that HRAS elicits protective effects in arthritis^43^. Therefore, we examined PLCη2–HRAS interactions at the single-cell level to verify their subcellular location. We constructed plasmids encoding recombinant PLCη2 and HRAS variants whose N- and C-termini were fused with the complementary halves of the split Venus fluorescent protein, either the N-terminal half (VN) or the C-terminal half (VC), which were cotransfected into MH7A cells (Fig. 6a). When the resulting proteins (VN-PLCη2 and VC-HRAS) were coexpressed in the same cells, strong fluorescence was detected in the cytosol (Fig. 6a). Furthermore, MH7A cells cotransfected with VN-PLCη2_C2 and VC-HRAS plasmids presented strong BiFC signals (Fig. 6a). However, when VN-PLCη2, VN-PLCη2_C2 and VC-HRAS were separately expressed, no fluorescence was detected (Fig. 6a). We also confirmed interactions between PLCη2 or PLCη2_C2 and HRAS in a mammalian system. We constructed vectors encoding GFP-tagged HRAS (GFP–HRAS) and HA-tagged PLCη2 and PLCη2_C2 (HA–PLCη2 and HA–PLCη2_C2, respectively), which were coexpressed in MH7A cells. We subsequently performed co-IP assays with cell lysates using anti-GFP and anti-HA antibodies for IP and IB analysis, respectively. We found that HA–PLCη2 strongly associated with GFP–HRAS (Fig. 6b, top), and another co-IP experiment revealed that HA–PLCη2_C2 interacted with GFP–HRAS (Fig. 6b, bottom). These results strongly suggest that associations between PLCη2 or PLCη2_C2 and HRAS occur in the cytosol of live mammalian cells. To verify the PLCη2 binding site with HRAS, mutant constructs of PLCη2 were generated. BiFC and co-IP assays were performed using full-length PLCη2 or C2 domain- or C2-deleted constructs to confirm the binding site between HRAS and PLCη2. The BiFC assay allows the visualization of two independent proteins in close spatial proximity. The BiFC data revealed that the weakest fluorescence was detected in cells cotransfected with HRAS and the PLCη2 C2 deletion mutant (ΔC2) (Supplementary Fig. 4a). Co-IP data revealed that HRAS strongly binds with the PLCη2 full-length or C2 domain but weakly interacts with C2-deleted PLCη2 (Supplementary Fig. 4b).

To further analyze signaling pathways downstream of PLCη2 in complex with HRAS, we measured RAS activity by performing RAS-GTP pulldown assays. Although the total levels of HRAS did not change in response to IL-1β or TG in PLCη2-overexpressing MH7A cells, the levels of active, GTP-bound HRAS decreased slightly (Fig. 6c). These results indicate that the PLCη2 protein interacts with HRAS and then inhibits RAF1 binding in RA FLSs. The RAF–MEK–ERK signaling axis was the first RAS effector described, and the original findings indicated that it regulated cell growth, inflammation and apoptosis^44^. Therefore, we studied the phosphorylation status of ERK, a kinase that acts downstream of activated RAS. Phosphorylated ERK (pERK) was detected by IB analysis using phospho-specific antibodies: increased pERK levels were detected in IL-1β- or TG-treated MH7A cells, whereas lower pERK levels were detected after GFP expression than after PLCη2 expression (Fig. 6c).

Next, we investigated the regulation of the intracellular signaling network resulting from PLCη2 using a phosphoproteome array. NF-κB-, JAK–STAT- and MAPK-related signaling pathways were examined in RA FLSs. Figure 6d shows that PLCη2 phosphorylation decreased in MH7A cells after IL-1β or TG treatment. Moreover, PLCη2 overexpression resulted in decreased phosphorylation levels in the downstream NF-κB, JAK–STAT and MAPK signaling pathways (Fig. 6d). By performing an NF-κB activity assay (Fig. 6e) and IB analysis (Fig. 6f) with IL-1β- and TG-treated MH7A cells, we confirmed that PLCη2 selectively regulated several components involved in signaling pathways important for RA FLS pathology. Overall, our results suggest that PLCη2 and PLCη2_C2 inhibited the proliferation and inflammatory responses of FLSs (which might be closely related to RA-associated intracellular signaling pathways) and that a functional link between PLCη2 and the HRAS–RAF1 protein complex may regulate PLCη2 activity in RA pathogenesis by directly inactivating the signaling pathway.

### PLCη2 and PLCη2_C2 exert therapeutic effects in mice with CIA

**Fig. 7:**
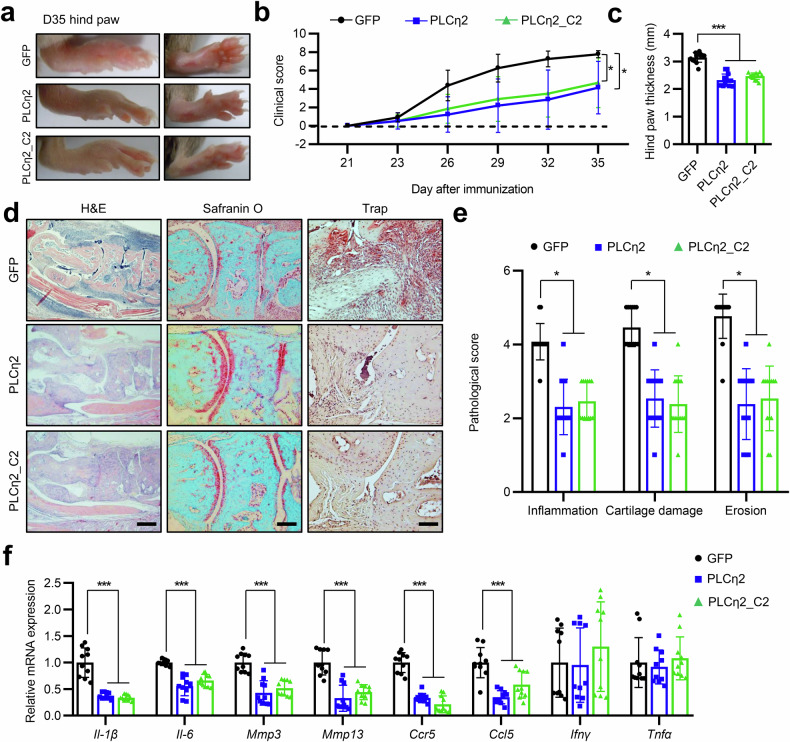
Anti-arthritic effects of PLCη2 and PLCη2_C2 in CIA mice. **a**–**f** Mice were injected with Ad-GFP, Ad-PLCη2 or Ad-PLCη2_C2 adenoviruses on day 20 (1 day before booster immunization on day 21). Gross images of the hind paw (**a**), the mean clinical score (**b**) and the ankle thickness (**c**) were obtained on the indicated days after the primary immunization. The hind paws of the mice with CIA were obtained on day 35 for histopathological analysis (**d**). The photomicrographs are representative of three independent experiments (40× magnification for H&E-stained sections, 100× magnification for Safranin-O-stained sections and 200× magnification for TRAP-stained sections). Synovial inflammation, cartilage damage and bone erosion were determined by H&E, safranin-O and TRAP staining (**e**), respectively. Transcript levels of various cytokines and/or chemokines (*Il-1β*, *Il-6*, *Mmp3*, *Mmp13*, *Ccr5*, *Ccl-5*, *Ifnγ* and *Tnf*) in the ankle joints (**f**). The bar graphs represent mean ± s.e.m. *P < 0.05, **P < 0.01, ***P < 0.001 versus the vector control (Ad-GFP) according to the Mann‒Whitney *U* test (**e**) and unpaired Student’s *t*-test (**f**).

Based on our in vitro (Fig. 4 and Supplementary Fig. 1) and in vivo data (Fig. 5) showing that PLCη2 and PLCη2_C2 dramatically inhibited the inflammatory and tumor-like behaviors of RA FLSs, we next assessed the possible therapeutic effects of PLCη2 and PLCη2_C2 on arthritis progression. For local delivery into the joint cavity, the ankles of mice with CIA were injected with 3 × 10^9^ PFUs of Ad-PLCη2, Ad-PCLη2_C2 or Ad-GFP 1 day before receiving a booster injection. The resulting pathological changes were observed after 35 days; the mice had swollen paws and ankles typical of arthritis (Fig. 7a). However, the clinical scores of the mice in the Ad-PLCη2 and Ad-PLCη2_C2 groups were significantly lower than those of the mice in the Ad-GFP group from day 35 (Fig. 7b). Hind paw swelling was measured to further analyze the beneficial effects of PLCη2 and PLCη2_C2 against CIA, and the results were consistent with the clinical scores (Fig. 7c). In addition, assessments of pathological features revealed that the ankle joints from Ad-GFP-injected mice with CIA exhibited synovial proliferation, cartilage damage, pannus formation and bone erosion (Fig. 7d, e). By contrast, the pathological findings of ankle joints from Ad-PLCη2- and Ad-PLCη2_C2-injected mice with CIA were markedly improved (Fig. 7d, e). We measured the mRNA expression levels of cytokines, MMPs and chemokines in mouse joint tissues using qRT‒PCR on day 35. We observed significantly lower levels of *Il-1β*, *Il-6*, *Mmp3*, *Mmp13*, *Ccr5* and *Ccl5* expression in the joint tissues of the Ad-PLCη2 and Ad-PLCη2_C2 groups than in those of the Ad-GFP group (Fig. 7f).

## Discussion

In this study, we demonstrated that PLCη2 was significantly associated with RA pathophysiology and was expressed at lower levels in RA FLSs than in OA FLSs. Our comprehensive in vitro and in vivo data convincingly revealed that PLCη2 and its C2 domain modulated the inflammatory and aggressive phenotypes of RA FLSs, thereby suppressing RA development and progression. Notably, we found that PLCη2 operates in a Ca^2+^-independent manner by studying protein–protein interactions within RA FLSs. We elucidated that PLCη2 and its C2 domain selectively bind to HRAS, suppressing its activity and, in turn, downregulating key downstream signaling pathways, including the MAPK–ERK, JAK–STAT and NF-κB pathways. These results identify PLCη2 as a potential upstream regulator with broad inhibitory effects and suggest its therapeutic potential in RA.

Several C2 domain-containing proteins, such as PKC^45,46^, phosphatase and tensin homolog (PTEN)^47,48^ and PI3K^49^, play pivotal roles in carcinogenic signaling. Consequently, targeting these proteins has become a cornerstone of new anticancer drugs^45,48,50^. These C2 domain-containing proteins, as well as molecules that modulate their expression levels or biological activities, are now being explored as novel treatments for a range of inflammatory or immune-mediated diseases^34,50,51^. However, the exact functions of many C2 domain-containing proteins remain unclear. Thus, comprehensive screening of a broad spectrum of C2 domain-containing proteins is crucial for identifying new therapeutic targets for various diseases. Furthermore, the phenotypic and functional diversity of RA FLSs^6^, along with the complex network of signaling pathways within these cells, necessitates methods for evaluating both quantitative differences and their functional roles. We used an innovative HTS-based, multiparametric, dynamic cellular assay targeting RA FLSs, leading to the identification of PLCη2_C2 as a therapeutic target from a pool of 144 C2 domains. PLCη2 was downregulated in the RA joint synovium, especially in RA FLSs. Our rigorous cellular and animal experiments revealed that increasing PLCη2 or PLCη2_C2 levels inhibited the inflammatory, hyperplastic, invasive and destructive behaviors of RA FLSs, potentially alleviating RA symptoms. These findings underscore the potential of PLCη2 and its C2 domain in RA treatment.

The unchecked growth of malignant and RA tissues can often be traced back to defects in the regulatory mechanisms driving cell proliferation and apoptosis^5,7,52^. As Ca^2+^ plays a crucial role in intracellular signaling, any disruption in the Ca^2+^ concentration and its binding to membrane phospholipids can significantly impact cell proliferation and apoptosis^37,38,53^. PLCη2, a recently identified C2 domain-containing protein, is particularly involved in regulating neuronal cells^54^. PLCη2 activity is initiated by the mobilization of intracellular Ca^2+^, which presumably enhances Ca^2+^ signaling by promoting additional Ca^2+^ release from the ER^55,56^. Our findings, however, indicate that, in RA FLSs, PLCη2 does not affect Ca^2+^ release from intracellular stores or influence the influx of extracellular Ca^2+^. Moreover, although other C2 domains in conventional PKCs and SYT1 can function as Ca^2+^-binding sensors to facilitate the translocation of C2 domain-containing proteins to the plasma membrane^35,57^, PLCη2 and its C2 domain do not promote membrane translocation in response to increased cytosolic Ca^2+^ in RA FLSs. These results indicate that PLCη2_C2 functions independently of Ca^2+^-mediated activities.

While many C2 domains are known for their Ca^2+^-binding capabilities^16,17^, others, such as those in the Munc13-1, KIBRA, PI3KC2α, RIM2, PTEN, SHIP2, Smurf1 and Smurf2 proteins, facilitate vital protein–protein interactions critical for intracellular signaling^58^. In this study, we identified HRAS as a PLCη2-binding partner and demonstrated that the interaction between PLCη2 and its C2 domain disrupted HRAS–RAF1 binding, leading to suppressed HRAS activity. HRAS, a member of the Ras family of GTPases, is frequently mutated in various cancers and the arthritic synovium, leading to the continuous activation of Ras proteins and initiating a cascade of intracellular signals that affect cell growth, differentiation and survival^43,44,59^. Consequently, therapeutic strategies targeting HRAS are being pursued in the fields of cancer and RA research^44,59,60^. Our findings revealed that PLCη2 directly interacted with HRAS, thereby inhibiting its function and consequently attenuating well-known RA-associated downstream signals, such as those in the NF-κB, JAK–STAT and MAPK pathways^44,52^.

RA is driven by complex interactions among immune cells, joint-resident FLSs and osteocytes involved in bone formation and resorption^1,2^. There is increasing interest in targeting RA FLSs to overcome the limitations of current immune cell-targeted drugs^5,61^. Various strategies are being developed to modulate signaling pathways, apoptosis, metabolism, surface markers and the epigenetic landscape of RA FLSs^50,52,61^. Notably, the use of JAK inhibitors has emerged as an important therapeutic modality for treating RA, unlike the observed clinical setbacks associated with p38 MAPK inhibitors^62^. JAK inhibitors have been demonstrated to successfully block upstream signaling, thereby interfering with multiple cytokine pathways simultaneously without inducing compensatory pathways^62^. Although it is difficult to rule out effects on synovial macrophages or immune cells after intraarticular injection of Ad-PLCη2 in CIA mice, the results of this study demonstrated that PLCη2 and its C2 domain bind to the key upstream signaling molecule HRAS, inhibiting its activity and broadly suppressing pathways such as the NF-κB, MAPK and JAK–STAT pathways, primarily in RA FLSs.

In conclusion, the C2 domain-containing protein PLCη2 was downregulated in RA FLSs, and augmenting PLCη2 or its C2 domain attenuated the inflammatory and aggressive traits of RA FLSs, thus hindering RA progression. Mechanistically, PLCη2 bound to and disrupted the activity of HRAS, a key upstream signaling molecule, resulting in the suppression of critical downstream signals associated with RA. These findings imply that both PLCη2 and its C2 domain, PLCη2_C2, are promising therapeutic targets for RA FLSs.

## Supplementary information

Supplementary Information

Supplementary Data

## Data Availability

The data generated and analyzed during this study are included in this Article and its Supplementary Information. Additional data are available from the corresponding author upon request.
