# Supplementary material for: The HRAS-binding C2 domain of PLCη2 suppresses tumor‐like synoviocytes and experimental arthritis in rheumatoid arthritis
Source: Exp Mol Med. 2025 Feb 3;57(2):335–48. doi: 10.1038/s12276-025-01393-5 (PMC11873285; doi:10.1038/s12276-025-01393-5)

Supplementary Information

The HRAS-binding C2 domain of PLC η 2 suppresses tumor‑like synoviocytes and experimental arthritis in rheumatoid arthritis

Hyun Min Jeon[1,4], Hae Sook Noh[1,4], Min-Gyu Jeon[1], Jin-Ho Park[1], Young-Sun Lee[2], Gyunghwa Seo[2], Yun-Hong Cheon[1], Mingyo Kim[1], Myung-Kwan Han[3], Jae-Yong Park[2*] and Sang-Il Lee[1*]

[1]Division of Rheumatology, Department of Internal Medicine and Institute of Medical Science, Gyeongsang National University School of Medicine and Gyeongsang National University Hospital, Jinju, South Korea; [2]School of Biosystem and Biomedical Science, College of Health Science, Korea University, Seoul, South Korea; [3]Microbiology, Jeonbuk National University Medical School, Jeonju, South Korea. [4]These authors contributed equally: Hyun Min Jeon, Hae Sook Noh.

This file includes:

1. Supplementary Materials & Methods:

2. Supplementary Figure and Figure legends:

Supplementary Fig. 1 to 4

3. Whole blots for Immunoblotting:

Whole immunoblots for Figure 3 (b and d)

Whole immunoblots for Figure 4 (a and f)

Whole immunoblots for Figure 6 (b, c and f)

Whole immunoblots for Supplementary Fig. 1c

Whole immunoblots for Supplementary Fig. 4b

1. Supplementary Materials & Methods:

Generating stable cell lines and performing transfections

To establish cell lines stably expressing PLCη2, a pcDNA3.1 encoding PLCη2 was transfected into MH7A cells using Lipofectamine 2000 (Invitrogen). At 3–4 weeks post-transfection, stable clones had been selected by culturing the cells in medium containing G418 sulfate (2.0 mg/mL, Thermo Fisher, USA). Stable PLCη2 expression was confirmed by IB analysis using an anti-PLCη2 antibody or detecting GFP expression under a fluorescence microscope. The clonal cell lines were subsequently maintained in 0.5 mg/mL G418 sulfate.

Immunoblotting (IB) and immunofluorescence (IF) staining analyses

RIPA buffer (Thermo Fisher, #89900) supplemented with protease and phosphatase inhibitors (Calbiochem, #535142 and Merck, #535142, respectively) was used to prepare cell lysates. The resulting lysates were sonicated and centrifuged, and the proteins in each sample were determined using the Bradford assay (Bio-Rad, USA, #500-0006), according to manufacturer's recommendations. Next, we added Laemmli Sample Buffer (Bio-Rad, #1610747) to equal volumes of protein, followed by denaturation of the lysates at 100 °C for 5 min. The samples (30–40 μg/lane) were then subjected to 8–12% SDS-PAGE and then transferred onto a nitrocellulose membrane (GE Healthcare, #GE10600002). The membrane was blocked with 5% non-fat milk in TBS-T buffer (containing 0.1% Tween-20) for 1 h. Subsequently, sections of the membrane corresponding to the expected molecular weights were stained overnight with primary antibodies against PLCη2 (1:1000;

MyBioSource, #MBS9609349), cPARP (1:1000; Cell Signaling Technology, #5625), Bax (1:1000; clone B-9, Santa Cruz Biotechnology, sc-7480), Bcl-2 (1:1000; clone N-19, Santa Cruz Biotechnology, sc-492), or β-actin (1:5000; Sigma-Aldrich, #A5441) prepared in TBS-T containing 1% milk. The blots were incubated with appropriate HRP-conjugated secondary antibodies (Bio-Rad, #170-6515 for anti-rabbit IgG; Bio-Rad, #170-6516 for goat anti-mouse) and developed with ClarityTM Western ECL Substrate (Bio-Rad, #170-5061). The densities of PLCη2 protein bands were normalized to the quantities of β-actin using Bio-Rad Image Lab software (version 6.0).

Double IF staining was performed using anti-human PLCη2 (1:40, Santa Cruz Biotechnology, sc-104624; S-16) and CD55 (1:200, Santa Cruz Biotechnology, sc-51733; NaM16-4D3) antibodies. As secondary antibodies, we used either an anti-mouse (for CD55) or anti-goat (for PLCη2) antibody (both diluted 1:500). Blocking was performed using PBS containing 10% fetal calf serum, and BSA for 40 min in a humidified chamber. The samples were incubated with primary antibodies (diluted in PBS containing 10% BSA) overnight at 4 °C. After washing, the samples were incubated with secondary antibodies (in PBS containing 10% BSA) for 2 h. After mounting with ProLong Gold Antifade Mountant with DNA Stain DAPI (Invitrogen, #P36931), the slides observed under a Nikon A1 fluorescence microscope. Fluorescence images were captured with Fluoview software.

qRT-PCR analysis

Total mRNA was extracted from frozen joint tissues or cells, using the Trizol reagent (Invitrogen) in accordance with the manufacturer’s instructions. Total RNA concentrations were determined using the QIAxpert System (Qiagen) and stored at -80 °C. For each

sample, 1 μg of total RNA was used to prepare cDNA using the iScript™ cDNA Synthesis Kit (Bio-Rad) according to the manufacturer's protocol. The expression levels of target genes were measured using a ViiA™ 7 Real-Time PCR System (Applied Biosystems Life Technologies). The following TaqMan Gene Expression Assays were used: Hs02758991_g1 for human *Gapdh*, Hs00988591_m1 for human *Plch2*, Hs00985639_m1 for human *Il-6*, Hs00968305_m1 for human *Mmp3*, Hs00233992_m1 for human *Mmp13*, Mm99999915_g1 for mouse *Gapdh*, Mm00434228_m1 for mouse *Il-1β*, Mm00446190_m1 for mouse *Il-6*, Mm00440295_m1 for mouse *Mmp3*, Mm00439491_m1 for mouse *Mmp13*, Mm01963251_s1 for mouse *Ccr5*, Mm01302427_m1 for mouse *Ccl5*, Mm01168134_m1 for mouse *Ifnγ*, and Mm00443258_m1 for mouse *Tnfα*. Real-time qPCR mixtures comprised 10 μL of Applied Biosystems™ TaqMan™ Gene Expression Master Mix, 1 μL of TaqMan assay, and 8 μL of ultra-pure water. For all reactions, a no-template control was also run, and the final reaction volumes were all 20 μL. The relative gene expression levels in each sample were determined using QuantStudio Real-Time PCR Software (version 1.2; Applied Biosystems Life Technologies). All amplifications were performed at least in triplicate. The relative quantities of the transcripts were calculated using the ΔΔCt method with the *Gapdh* expression serving as an endogenous control, as described by the manufacturer (Applied Biosystems Life Technologies).

Wound-healing and Transwell migration/invasion assays

For wound-healing and transwell migration/invasion assay, RA FLSs, grown to 70% confluence on 35-mm culture dishes, were serum-starved for 12 to 16 h and wounded with 200 μL pipette tips. The culture dishes were washed three times with PBS to remove the

detached cells, and the remaining cells were grown in DMEM containing 10% FBS. After 48 h of incubation, migration was quantified by counting the cells that had moved beyond a reference line.

Transwell cell migration/invasion assays were performed to measure the chemotactic capability of cells toward a chemoattractant. Chemotaxis assays were performed with FLSs using the Boyden chamber method with 6.5 mm filters (pore size: 8.0 μm; Transwell, Corning Labware Products, #3422). Briefly, DMEM containing 10% FBS or 10 ng/mL IL-1β was placed as a chemoattractant in the lower wells, and suspended FLSs (6×10^4 cells/mL) were placed in serum-free DMEM in the upper wells. The plates were incubated for 12 h at 37°C in 5% CO_2. Subsequently, the non-migrating cells were removed from the filter's upper surface using a cotton swab. The filters were fixed in methanol for 15 min and stained with DAPI for 15 min. Chemotaxis was quantified using an optical microscope to count the stained cells that had migrated to the lower side of the filter. The stained cells were counted as the mean number of cells in three random fields for each assay. For the in vitro invasion assays, similar experiments were conducted using inserts coated with BD Matrigel Basement Membrane Matrix (BD Biosciences) and DMEM containing 10% FBS as a chemoattractant. The plates were incubated for 48 h at 37 °C in 5% CO_2.

Measuring intracellular Ca^{2+} concentrations

To determine whether changes in intracellular Ca^{2+} concentration were due to Ca^{2+} release from the Ca^{2+}-storing organelles or Ca^{2+} influx from outside of the cells, experiments were conducted in Ca^{2+}-free solution. Intracellular Ca^{2+} stores were depleted with TG and

subsequently, 2 mM Ca^{2+} was added to the medium to reveal Ca^{2+} influx via SOCE. The cells were cultured on confocal dishes for 3 h, washed with HBSS (2 mM $CaCl_2$, 145 mM NaCl, 5 mM KCl, 1 mM $MgCl_2$, 5 mM D-glucose, and 10 mM HEPES, pH 7.4), and then loaded with 20 μM Fura-2 AM (Sigma) for 1 h. Before stimulation, the cells were held at 37 °C for 1 min, and intracellular Ca^{2+} responses were then induced with TG in Ca^{2+}-free solution. Subsequently, changes in the Ca^{2+} concentrations were observed. After 2–3 min, the cells were treated with 2 mM $CaCl_2$, and further changes in the Ca^{2+} concentrations were monitored. The changes in the intracellular Ca^{2+} concentrations were determined by measuring the fluorescence intensities of Fura-2 AM using dual excitation wavelengths of 340 and 380 nm and an emission wavelength of 510 nm with a fluorescence microscope. The data were analyzed using the MetaFluor program (Molecular Devices). The results are presented as the ratio of fluorescence at 340 nm to that at 380 nm.

2. Supplementary Figures and Figure legends:

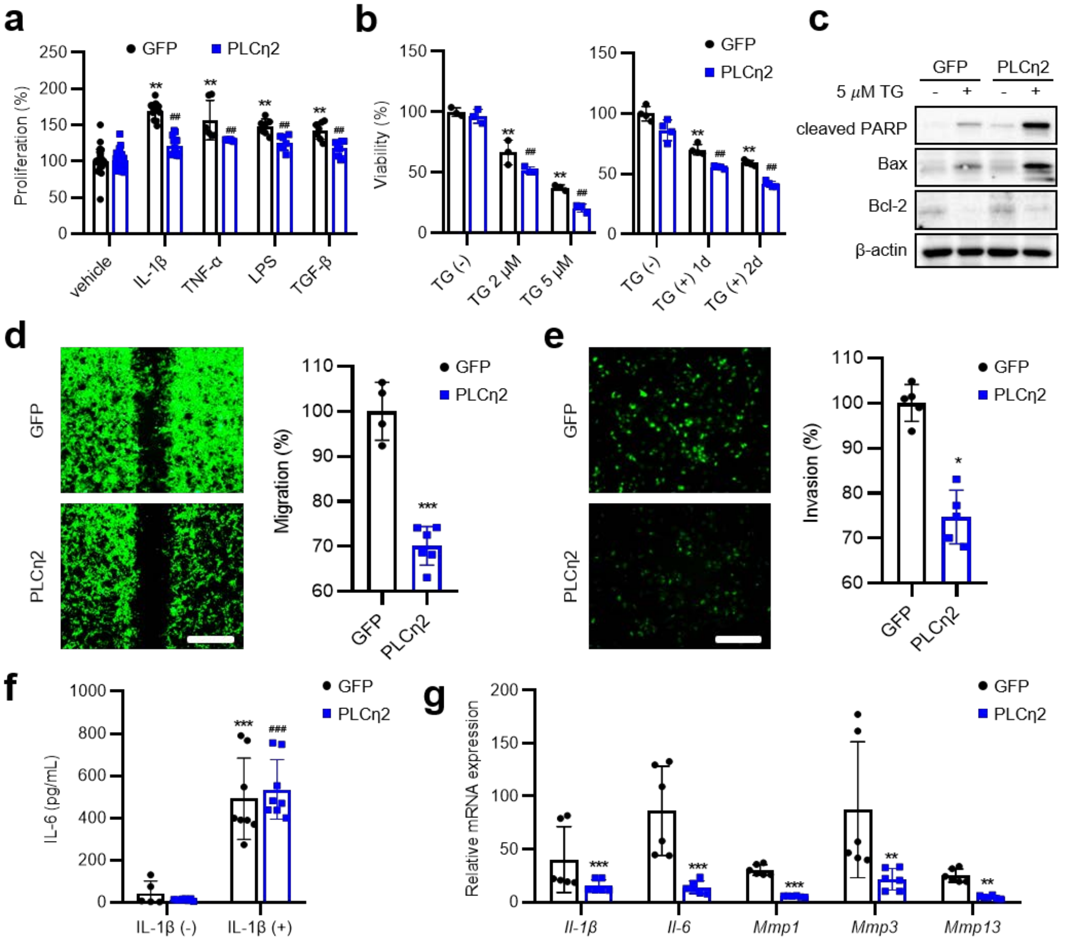

Supplementary Fig. 1 Effects of full-length PLCη2 against aggressive and inflammatory phenotypes of MH7A cells. a MH7A cells stably transfected with a GFP- or PLCη2-expression vector were incubated with various agents (20 ng/mL IL-1β, 50 ng/mL TNF-α, 10 μg/mL LPS, or 1 ng/mL TGF-β), and cell proliferation was then assessed by performing CCK-8 assays. The results are expressed as percentages relative to the data obtained with the vector control (Ad-GFP). **b** MH7A cells stably transfected with a GFP- or PLCη2-expression vector were treated with TG under the indicated conditions, and cell viabilities were analyzed by performing MTT assays. **c** Immunoblots of stable GFP- or PLCη2-overexpressing MH7A cell lines treated with TG for 48 h. **d, e** Effect of PLCη2

overexpression on the migration and invasion of MH7A cells. Representative images are shown (original magnification, ×40). The relative migration (**d**) and invasion (**e**) data shown represent the mean ± SEM of four independent experiments involving samples from four different patients with RA. **f** Quantification of IL-6 serum levels in PLCη2-overexpressing MH7A cells during IL-1β stimulation. **g** mRNA-expression levels of pro-inflammatory factors in PLCη2-overexpressing MH7A cells were determined via RT-qPCR. The graphs indicate the mean ± SEM of three independent experiments. $^{*}P < 0.05$, $^{**}P < 0.01$, and $^{***}P < 0.001$, versus control (vehicle, vector control); $^{\#\#}P < 0.01$ and $^{\#\#\#}P < 0.001$ versus GFP as determined by one-way ANOVA followed by unpaired, 2-tailed t test (**a, b, f** and **g**) and unpaired Student's t-test (**d, e**). Comparison of numerical data between groups were performed using the unpaired t test, Welch's t test, Mann-Whitney U test, or Tukey's multiple comparisons test.

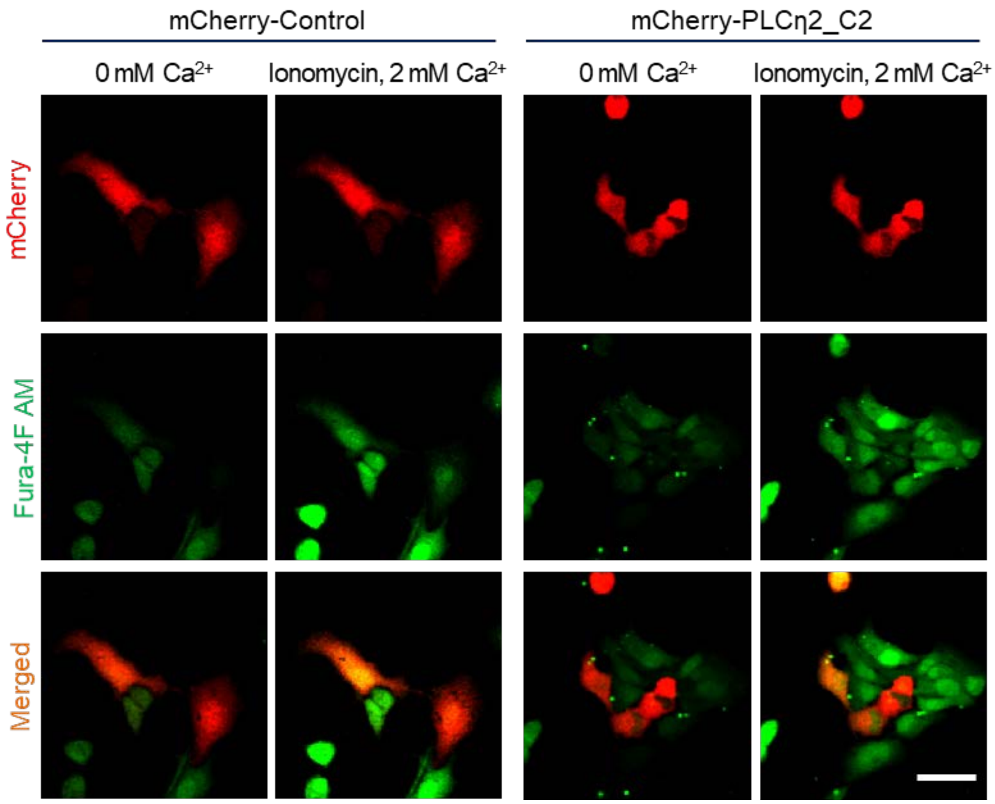

Supplementary Fig. 2 The C2 domain did not undergo plasma membrane translocation in response to ionomycin-induced Ca^{2+} release. Confocal microscopy images of MH7A cells co-transfected with mCherry plasmids and PLCη2_C2 plasmid or empty plasmid. The data show mCherry expression (red), Fura-4 AM fluorescence (green), and merged channels. Ionomycin (10 μM) was added to trigger store Ca^{2+} depletion. The data shown are representative of at least three different experiments. Magnification: 10×; scale bar, 50 μm

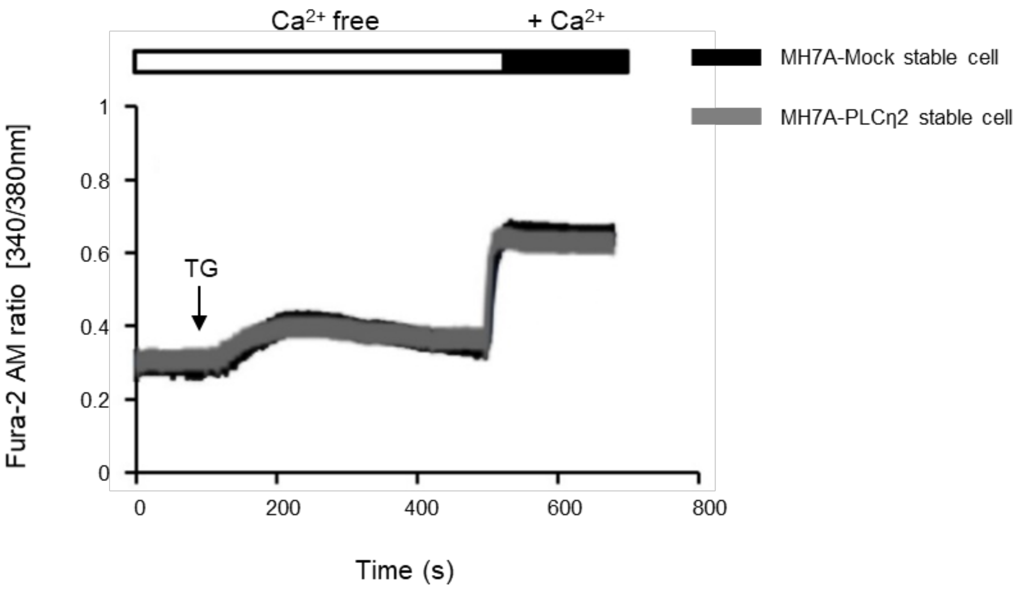

Supplementary Fig. 3 Effect of PLCη2 on Ca^{2+} release and entry in MH7A cells. MH7A cells stably transfected with a Mock (black) or PLCη2 (grey) plasmid were labeled with the intracellular Ca^{2+}-indicator Fura-2 AM and then stimulated with TG. The intracellular Ca^{2+} concentration was determined at 37 °C by monitoring changes in Fura-2 AM fluorescence. Cells pre-loaded with Fura-2 AM were analyzed via time-lapse ratiometric Ca^{2+} imaging (340/380 nm excitation ratio). The mean of three independent experiments is shown.

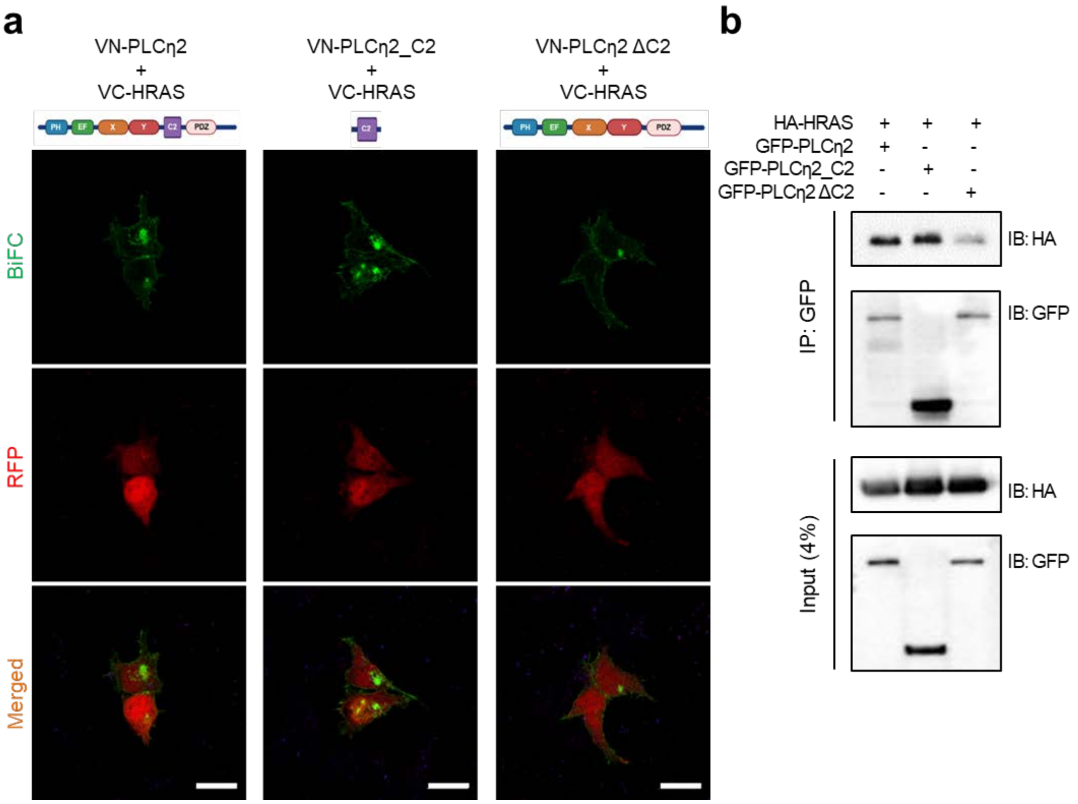

Supplementary Fig. 4 Interaction of PLCη2 and PLCη2_C2 on HRAS. a Confocal images of BiFC assay. VC-HRAS and VN-PLCη2 full length (VN-PLCη2), VN-PLCη2 C2 domain (VN-PLCη2_C2), VN-PLCη2 C2 deletion mutant (VN-PLCη2 ΔC2) were transfected in HEK293T. GFP: BiFC signal, RFP: transfection control, Scale bar; 20 µm. **b** Co-IP analysis of the interaction between HRAS and PLCη2 or PLCη2 C2 domain or PLCη2 C2 deletion mutant in HEK293T.

3. Whole blots for Immunoblotting:

Whole immunoblots for Figure 3

Fig. 3b

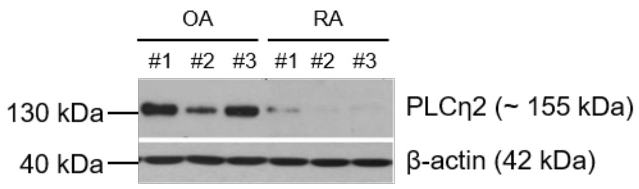

Fig. 3d

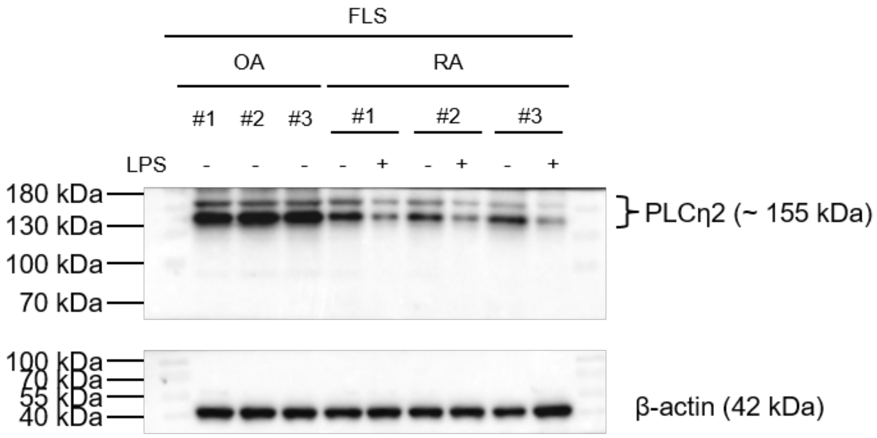

Whole immunoblots for Figure 4

Fig. 4a

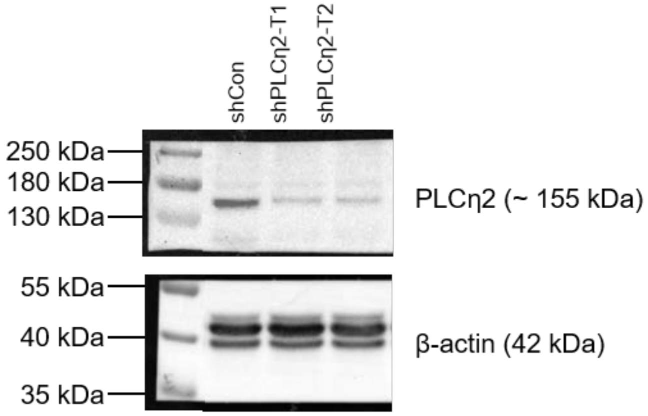

Fig. 4f

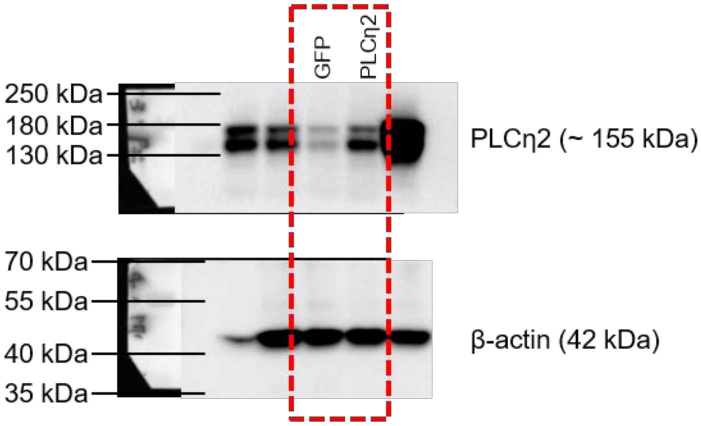

Whole immunoblots for Figure 6

Fig. 6b

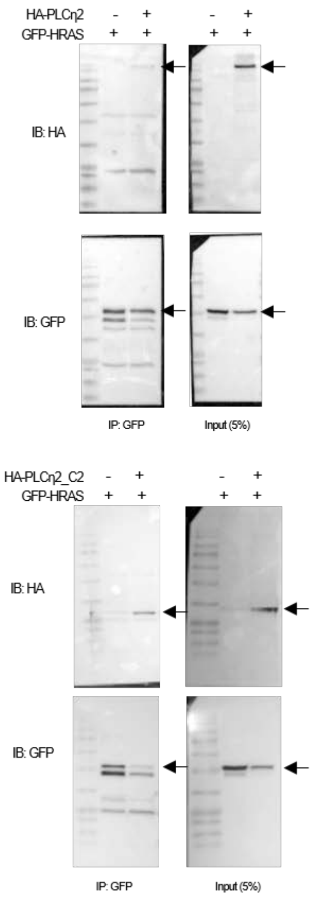

Fig. 6c

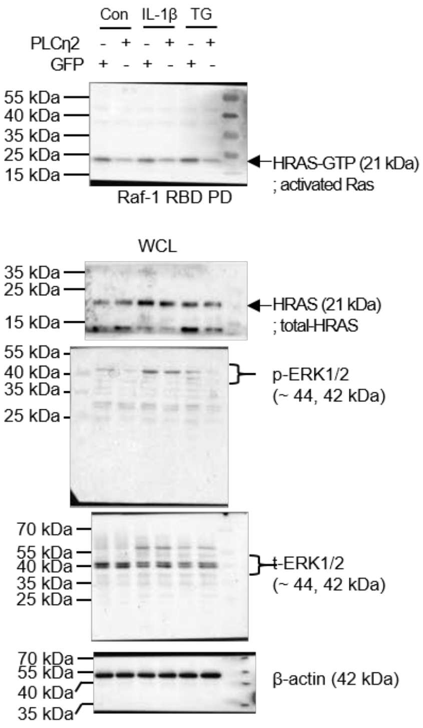

Fig. 6f

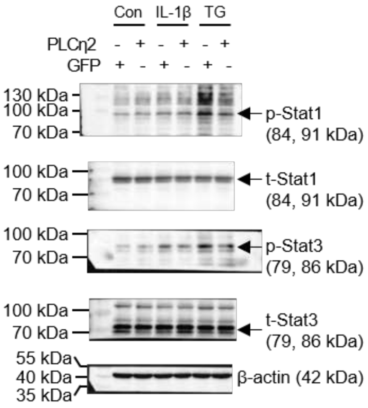

Whole immunoblots for Supplementary Fig. 1c

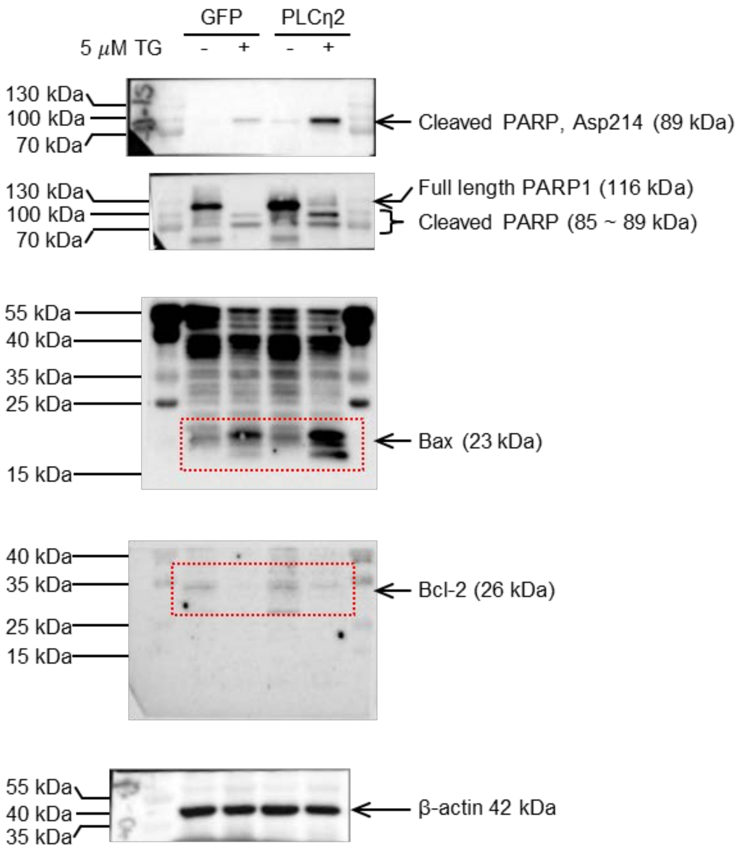

Whole immunoblots for Supplementary Fig. 4b

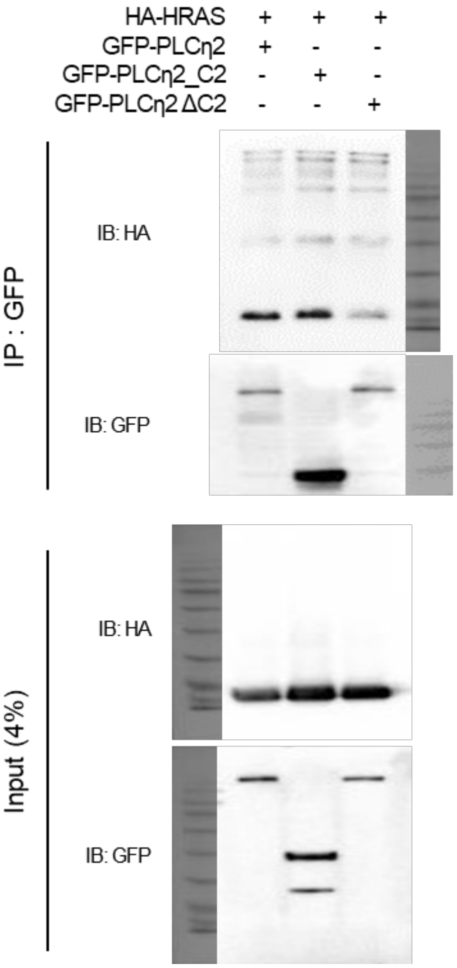

Supplement: Supplementary file 1 — Supplementary Information [file 12276_2025_1393_MOESM1_ESM.pdf]
